# Effect of HIV-1 Tat on the formation of the mitotic spindle by interaction with ribosomal protein S3

**DOI:** 10.1038/s41598-018-27008-w

**Published:** 2018-06-06

**Authors:** Jiyoung Kim, Yeon-Soo Kim

**Affiliations:** Graduate School of New Drug Discovery and Development, Chungnam National University, 99 Daehak-ro, Yusung-gu, Daejeon, 34134 South Korea

## Abstract

Human immunodeficiency virus type 1 (HIV-1) Tat, an important regulator of viral transcription, interacts with diverse cellular proteins and promotes or inhibits cell proliferation. Here, we show that ribosomal protein S3 (RPS3) plays an important role in mitosis through an interaction with α-tubulin and that Tat binds to and inhibits the localization of RPS3 in the mitotic spindle during mitosis. RPS3 colocalized with α-tubulin around chromosomes in the mitotic spindle. Depletion of RPS3 promoted α-tubulin assembly, while overexpression of RPS3 impaired α-tubulin assembly. Depletion of RPS3 resulted in aberrant mitotic spindle formation, segregation failure, and defective abscission. Moreover, ectopic expression of RPS3 rescued the cell proliferation defect in RPS3-knockdown cells. HIV-1 Tat interacted with RPS3 through its basic domain and increased the level of RPS3 in the nucleus. Expression of Tat caused defects in mitotic spindle formation and chromosome assembly in mitosis. Moreover, the localization of RPS3 in the mitotic spindle was disrupted when HIV-1 Tat was expressed in HeLa and Jurkat cells. These results suggest that Tat inhibits cell proliferation via an interaction with RPS3 and thereby disrupts mitotic spindle formation during HIV-1 infection. These results might provide insight into the mechanism underlying lymphocyte pathogenesis during HIV-1 infection.

## Introduction

Human immunodeficiency virus type 1 (HIV-1) Tat is an important regulator of viral transcription. The primary role of Tat is transactivation of the HIV-1 long-terminal repeat promoter, which is essential for viral replication^1^. In addition, HIV-1 Tat is involved in various cellular processes including the regulation of translation^2,3^, induction of angiogenesis^4^, modulation of cytokine expression^5^, and activation of cellular signaling pathways^6^.

HIV-1 Tat promotes or inhibits host cell growth by regulating cellular proteins. Downregulation of tyrosine phosphorylation by HIV-1 Tat inhibits growth of Kaposi’s sarcoma-like spindle cells^7^. HIV-1 Tat-mediated induction of platelet-derived growth factor increases proliferation of astrocytes^8^. HIV-1 Tat interacts with tubulin and this leads to alteration of microtubule dynamics, facilitating apoptosis^9^. Injection of recombinant Tat into *Drosophila* syncytial embryos prolongs the time taken for kinetochore alignment and exit from mitosis. Furthermore, expression of Tat in *Drosophila* larvae brain cells significantly increases the number of aneuploid and polyploid cells, suggesting an important role for Tat in mitosis^10^. A recent study using recombinant Tat suggests that Tat interacts with Eg5, a microtubule-associated motor, and contributes to activation of the mitotic spindle checkpoint^11^.

Ribosomal protein S3 (RPS3) is a component of the 40S ribosome and has various extra-ribosomal functions. RPS3 is involved in DNA repair by cleavage of DNA at apurinic/apyrimidinic sites of DNA damage^12^ or by processing of 8-oxoguanine DNA lesions produced during oxidative stress^13^. The DNA repair activity of RPS3 is dependent on its translocation into the nucleus, which is governed by phosphorylation of the protein by cellular kinases such as protein kinase Cδ^14^ or extracellular signal-regulated kinase 1^15^. Overexpression of RPS3-GFP induces chromosome condensation and promotes the degradation of poly (ADP-ribose) polymerase, suggesting it has a role in apoptosis^16^. Caspase-3, -8, and -9 are activated by overexpression of RPS3, indicating that RPS3-mediated apoptosis is caspase-dependent^17^. Interestingly, apoptosis induction by RPS3 is also regulated by phosphorylation; phosphorylation of RPS3 by Akt kinase inhibits its apoptotic function^16^. Accumulating data suggest that RPS3 plays a role in microbial pathogenesis. The bacterial protein NleH1 inhibits the translocation and phosphorylation of RPS3 that is required to guide NFκB to specific κB sites and therefore to promote the expression of proteins involved in the immune response^18^. Recent studies showed that depletion of RPS3 inhibits melanoma tumor growth^19^ or osteosarcoma invasion^20^, suggesting that it has a role in cell proliferation. RPS3 localizes to the mitotic spindle and mitotic arrest is induced in RPS3-depleted cells, indicating that RPS3 plays a role in mitosis and regulation of cell growth^21^.

Here, we show that that RPS3 plays an important role in mitosis through an interaction with α-tubulin, while Tat inhibits cell proliferation by interacting with and disturbing the localization of RPS3 in the mitotic spindle during mitosis. Knockdown of RPS3 results in aberrant mitotic spindle formation, segregation failure, and defective abscission. Moreover, RPS3 interacts with α-tubulin in G2/M phase of the cell cycle and depletion of RPS3 impairs microtubule disassembly. HIV-1 Tat interacts with RPS3 via its basic domain and increases the nuclear level of RPS3. Expression of Tat causes defects in mitotic spindle formation and chromosome assembly as well as the aberrant distribution of RPS3 in the mitotic spindle during mitosis in both HeLa and Jurkat cells. These results might provide insight into the mechanism underlying lymphocyte pathogenesis during HIV-1 infection.

## Results

### HIV-1 Tat interacts with RPS3 through its basic domain

**Figure 1:**
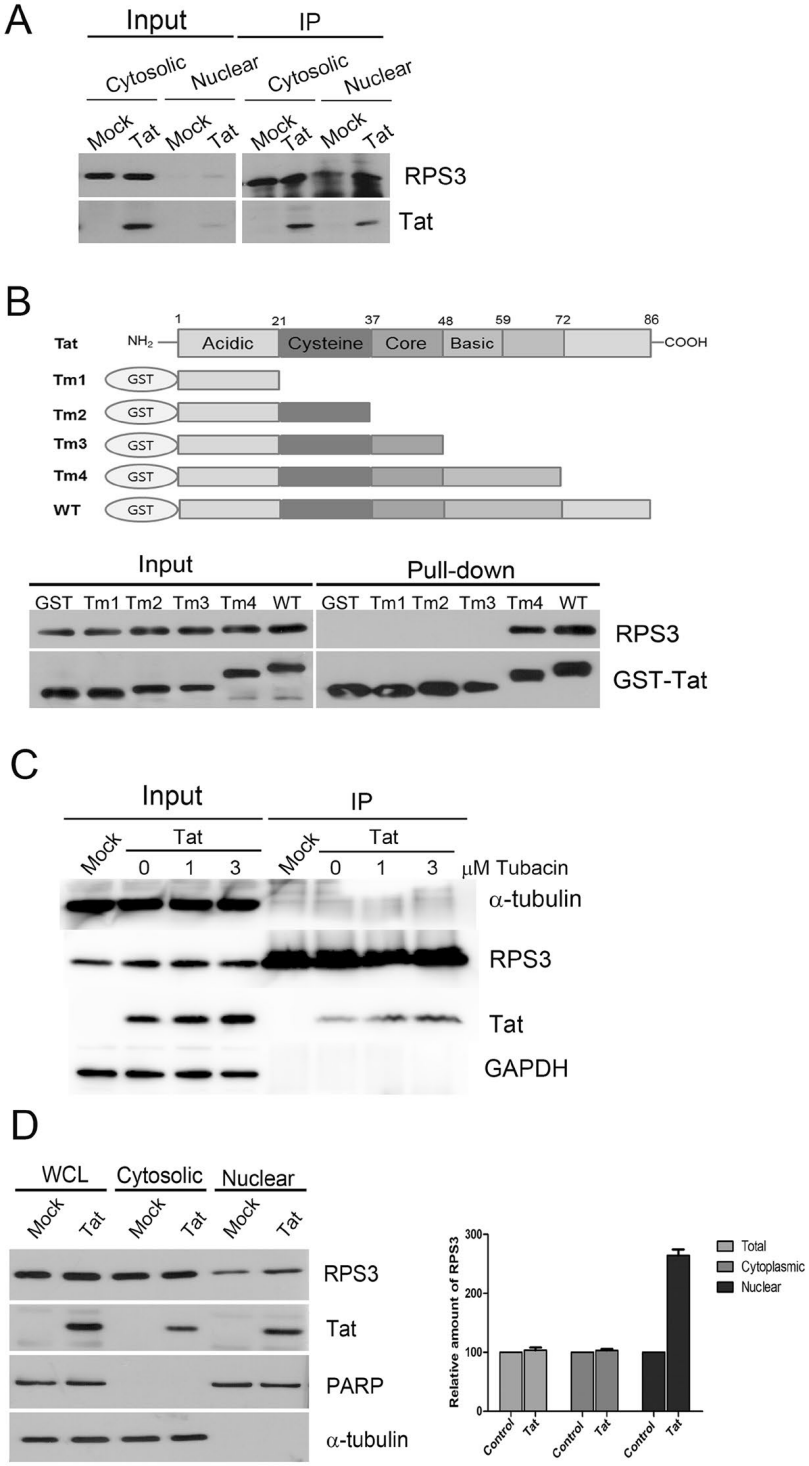
HIV-1 Tat interacts with RPS3 and induces nuclear accumulation of RPS3. (**A**) Co-immunoprecipitation of Tat and RPS3 in HEK 293FT cells transfected with the control or Tat expression construct. Results are representative of three independent experiments. (**B**) Tat binds to RPS3 through its basic domain. Expression constructs encoding GST-Tat deletion mutants were transfected into 293FT cells, GST-Tat deletion mutants were pulled down with glutathione agarose beads, and co-precipitation of RPS3 was examined by immunoblotting. (**C**) Acetylation of Tat at K28 may increase its interaction with RPS3. 293FT cells were transfected with the Tat expression construct and incubated for 24 h in the presence of 0, 1, or 3 μM tubacin, an HDAC6 inhibitor. Thereafter, 500 μg of each cell extract was subjected to immunoprecipitation with an anti-RPS3 antibody. Bound proteins were analyzed by immunoblotting with anti-Tat, anti-α-tubulin, anti-GAPDH, and anti-RPS3 antibodies. (**D**) HIV-1 Tat induces nuclear accumulation of RPS3 in 293FT cells. 293FT cells were transfected with the Tat expression construct and incubated for 16 h. Nuclear and cytosolic fractions were separated and analyzed by western blotting with anti-α-tubulin, anti-PARP, anti-RPS3, and anti-Tat antibodies (left panel). WCL: whole cell lysate. Blots were scanned and the intensity of the RPS3 band was quantified by ImageJ software and normalized against that in the control sample. Data represent the mean ± SEM of four independent experiments (right panel).

During efforts to identify cellular proteins that interact with Tat (strain HIV-BRU, 86 amino acids), RPS3 was isolated as a potential Tat-binding protein in matrix-assisted laser desorption time-of-flight mass spectrometry. To confirm the interaction between Tat and RPS3, 293FT cells were transfected with a Tat expression construct and incubated for 16 hr. Cytosolic and nuclear fractions were separated and subjected to immunoprecipitation with an anti-RPS3 antibody, and co-precipitation of Tat was examined by immunoblotting. A large amount of Tat protein co-precipitated with RPS3 in both cytosolic and nuclear fractions, while no interaction was detected in mock-transfected cells (Fig. 1A). Cropped blots are shown in Fig. 1A, and full-length blots are presented in Supplementary Fig. S6. In a reverse immunoprecipitation assay with an anti-Tat antibody, a large amount of RPS3 were co-precipitated with Tat (see Supplementary Fig. S1). However, a marked amount of RPS3, as well as RPS6 and RPL26, was also precipitated with normal serum, indicative of non-specific binding of the ribosome complex to normal serum. Both RPS6 and RPL26 co-precipitated with RPS3 upon immunoprecipitation with an anti-Tat antibody, suggesting that ribosomal proteins co-precipitated with Tat due to its interaction with RPS3.

Tat is composed of five different functional domains that play important roles in transactivation (cysteine-rich domain), nuclear localization, and RNA binding (basic domain) (Fig. 1B). To determine which region of Tat is involved in the interaction with RPS3, we constructed serial C-terminal deletion mutants of Tat in the form of GST-fusion proteins. Expression constructs encoding the GST-Tat deletion mutants were transfected into 293FT cells, GST-Tat deletion mutants were pulled down with glutathione agarose beads, and co-precipitation of RPS3 was examined by immunoblotting. RPS3 bound to Tat containing the basic domain but not to the core domain, suggesting that the basic domain of Tat is important for RPS3 binding (Fig. 1B). Cropped blots are shown in Fig. 1B and full-length blots are presented in Supplementary Fig. S7.

Huo *et al*. reported that acetylation of Tat at K28 modulates its effects on microtubule dynamics, which are critical for spindle formation and mitosis^22,23^. To examine the effect of the acetylation of Tat on interaction with RPS3, tubacin, a specific inhibitor of HDAC6, was used to inhibit the acetylation of Tat at K28. RPS3 was precipitated with an anti-RPS3 antibody, and co-precipitated Tat was analyzed by immunoblotting. Tubacin treatment increased the amount of Tat associated with RPS3 in a dose-dependent manner. In addition, tubacin treatment also increased the level of Tat in the input in a dose-dependent manner (Fig. 1C). Acetylation is known to increase the stabilities of some proteins^24^. Therefore, the increased level of Tat upon tubacin treatment might be due to increase of stability of this protein. These results suggest that acetylation of Tat at K28 enhances its association with RPS3 by increasing its stability. Tubacin treatment did not increase the level of α-tubulin associated with RPS3 (Fig. 1C). Cropped blots are shown in Fig. 1C and full-length blots are presented in Supplementary Fig. S8.

To determine whether Tat interacts directly with RPS3 or the interaction is mediated by another component of the ribosome complex, the localizations of RPL26, a component of the 60S ribosome complex, and RPS6, a component of the 40S ribosome complex, were examined in the presence of Tat. Different from RPS3, the nuclear levels of RPS6 and RPL26 did not increase in the presence of Tat, suggesting that the increased nuclear level of RPS3 is dependent on its interaction with Tat (see Supplementary Fig. S2).

The nuclear level of RPS3 in the presence of Tat was examined through subcellular fractionation of 293FT cells. Cytosolic and nuclear fractions were separated from mock- or Tat-transfected 293FT cells, and the level of RPS3 in the presence or absence of Tat was examined by immunoblotting. Expression of Tat did not increase the total level of RPS3, ruling out the possibility that the increased nuclear level of RPS3 was caused by the transactivation activity of Tat (Fig. 1D, right panel). The majority of RPS3 protein was localized in the cytosol and only a low level of RPS3 was found in the nucleus in mock virus-infected cells. The nuclear level of RPS3 was increased in the presence of Tat, suggesting that Tat recruited RPS3 into the nucleus (Fig. 1D, left panel). Cropped blots are shown in Fig. 1D and full-length blots are presented in Supplementary Fig. S9.

### RPS3 localizes to the mitotic spindle during mitosis

Because RPS3 is a component of the ribosome complex, we reasoned that the interaction between Tat and RPS3 might regulate cell proliferation. As a first step to examine this possibility, proliferation of RPS3-depleted cells was examined. 293FT cells were transduced with a lentivirus expressing the control or RPS3 shRNA construct (shRPS3). The shRPS3 efficiently suppressed the expression of RPS3 protein (see Supplementary Fig. S3A), and prolonged exposure to shRPS3 inhibited the growth of 293FT cells (see Supplementary Fig. S3B,C). The shRPS3-4 showed most efficient knockdown of RPS3 and inhibited cell growth, and was thereafter used to knockdown RPS3 in subsequent experiments. Next, the nuclear localization of Tat was examined in RPS3-knockdown cells to examine whether the localization of Tat in the nucleus is dependent on its interaction with RPS3. 293FT cells were transduced with the shRPS3-4, transfected with a Tat expression construct, and stained with anti-RPS3 and anti-Tat antibodies as well as DAPI. Tat protein was accumulated in the nuclei of shRPS3-4-treated cells, suggesting that knockdown of RPS3 does not affect the nuclear localization of Tat (see Supplementary Fig. S3D). Interestingly, RPS3-knockdown cells had an abnormal nuclear morphology with an aberrant and deformed structure compared with control virus-infected cells, suggesting that there are some dysfunctions in the regulation of mitosis (see Supplementary Fig. S3E).

**Figure 2:**
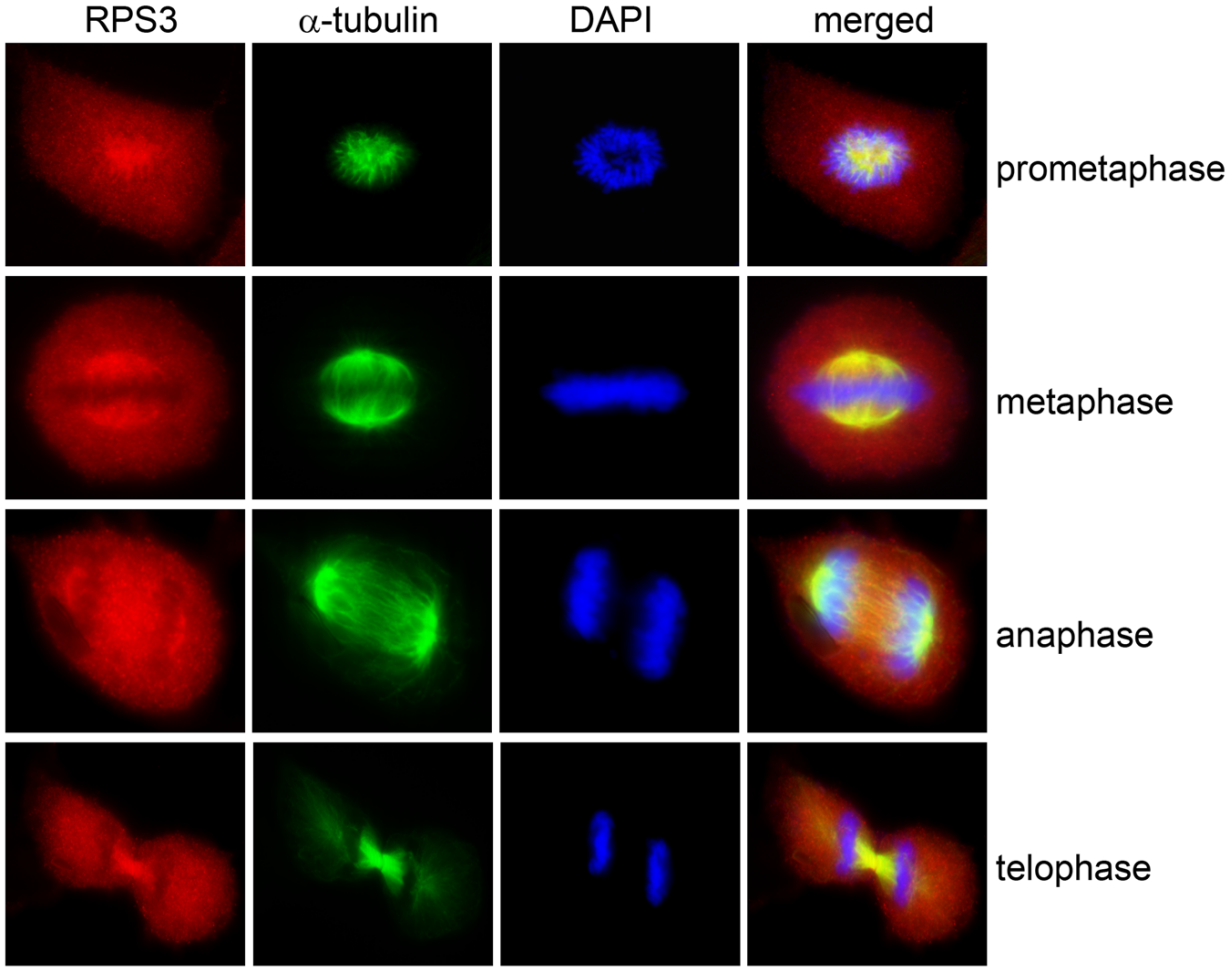
RPS3 localizes in the mitotic spindle during mitosis. Fluorescence microscopy images of HeLa cells immunostained with anti-RPS3 and anti-α-tubulin antibodies are shown. RPS3 colocalized with α-tubulin throughout mitosis. n = 86. Experiments were repeated three times.

To determine whether RPS3 is involved in mitosis, HeLa cells were immunostained with anti-RPS3 and anti-α-tubulin antibodies. RPS3 colocalized with α-tubulin throughout mitosis, suggesting that RPS3 plays a role in mitotic spindle formation. In prometaphase, RPS3 was assembled in the center of cells and colocalized with α-tubulin, and chromosomes were arrayed around RPS3. In metaphase, RPS3 was concentrated around chromosomes and colocalized with α-tubulin and the proteins were in the mitotic spindle. In anaphase, RPS3 was concentrated at the spindle pole, but a significant amount of RPS3 was found in the spindle midzone. In telophase, RPS3 was localized in newly formed nuclei and the midbody (Fig. 2). When HeLa cells were stained with an anti-RPS3 antibody only, RPS3 showed the same pattern as in cells stained with anti-RPS3 and anti-α-tubulin antibodies (see Supplementary Fig. S4A). To rule out the possibility that the anti-RPS3 antibody used in this experiment bound to α-tubulin non-specifically, the pmCherry-rps3 plasmid was transfected into HeLa cells and the localization of RPS3 in mitotic cells was examined. mCherry-RPS3 was concentrated around chromosomes in metaphase (see Supplementary Fig. S4B), suggesting that RPS3 does indeed localize in the mitotic spindle during mitosis. Co-staining of HeLa cells with anti-RPS3 and anti-RPS6 antibodies showed the localization of RPS3 and RPS6 around chromosomes in metaphase, but only RPS3 was localized in the mitotic spindle pole (see Supplementary Fig. S4C). This result suggests that RPS3, but not the 40S ribosome complex, plays a role in mitotic spindle formation.

### Knockdown of RPS3 results in aberrant mitotic spindle formation, segregation failure, and defective abscission

**Figure 3:**
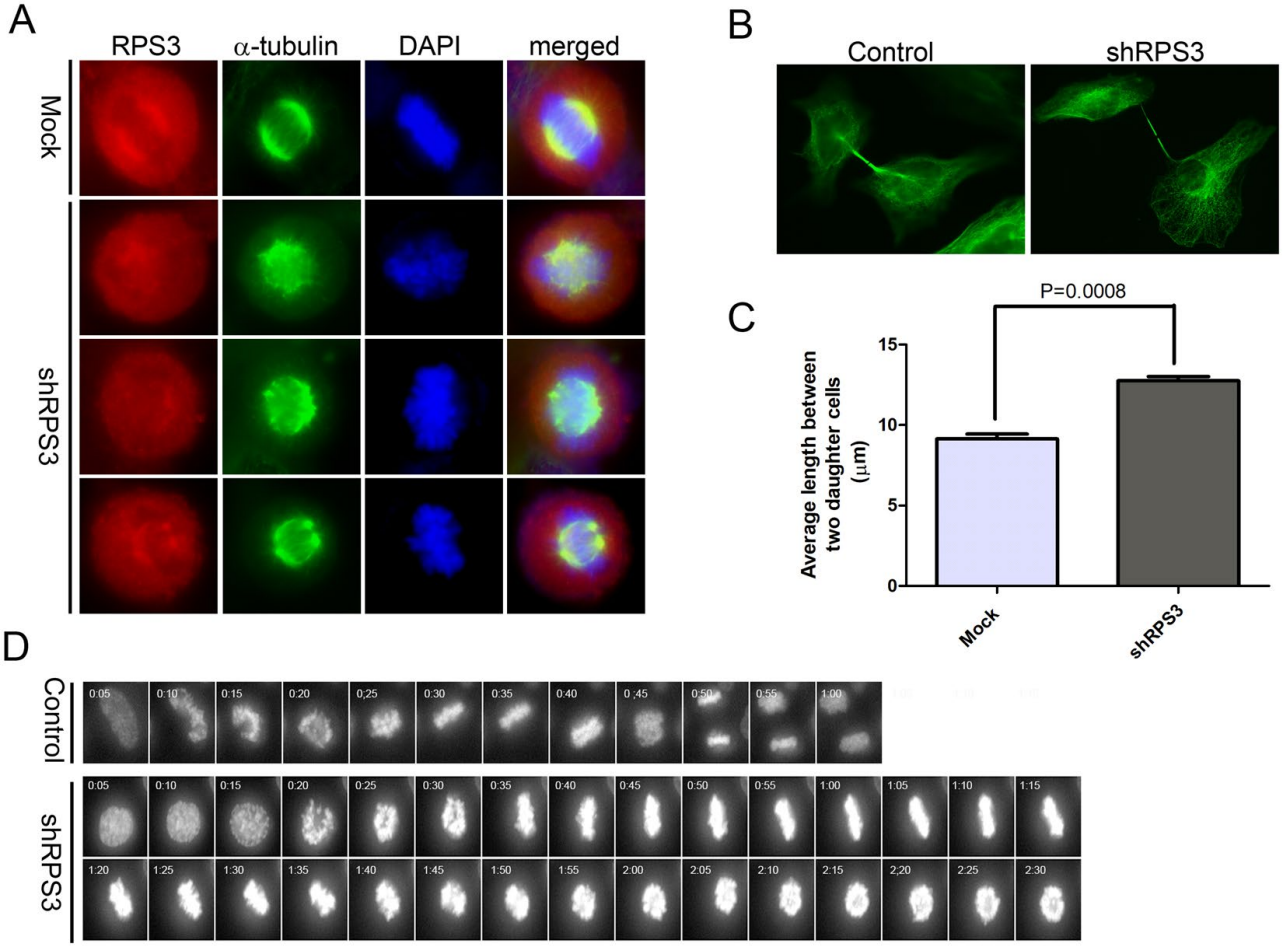
Knockdown of RPS3 results in aberrant mitotic spindle formation, segregation failure, and defective abscission. (**A**) Aberrant mitotic spindle formation and segregation failure in RPS3-depleted cells. HeLa cells were transduced with shRPS3-4 and stained with anti-α-tubulin and anti-RPS3 antibodies. Control, n = 71; shRPS3, n = 112. Experiments were repeated three times. (**B**) Transduction of shRPS3 resulted in a defect in abscission. HeLa cells were transduced with shRPS3-4 and stained with an anti-α-tubulin antibody. (**C**) The average length of bridges between two daughter cells is plotted for control and RPS3-knockdown cells. Data represent the mean ± SEM of three independent experiments. Statistical analysis was performed using the two-tailed *t* test. Control, n = 356; RPS3-knockdown, n = 356. P = 0.0008. (**D**) Time-lapse images of control and shRPS3-4-transduced HeLa-H2B-GFP cells. HeLa-H2B-GFP cells were transduced with the control or shRPS3-4 virus and incubated for 2 days. Cells were plated onto chamber slides and incubated for a further 24 h. Images were acquired using a 40×, NA 0.6 dry objective lens every 5 min in a chamber at 37 °C in 5% CO_2_ and processed using ImageJ software.

The role of RPS3 in mitosis was further examined in RPS3-depleted cells. HeLa cells were infected with shRPS3-4, and the localization of RPS3 in mitosis was examined by immunostaining with anti-RPS3 and anti-α-tubulin antibodies. RPS3 protein was still concentrated around chromosomes during mitosis in RPS3-knockdown cells. However, the distribution of RPS3 in the mitotic spindle showed various defects, including longer, denser, and misaligned RPS3 and multipolar RPS3 (Fig. 3A). α-Tubulin colocalized with RPS3 in these cells, resulting in defective mitotic spindles. In late telophase, two daughter cells were completely separated by abscission of the midbody in control HeLa cells (Fig. 3B). However, the two daughter cells were not separated and remained linked together by a thin and long stretch of the spindle in many RPS3-knockdown cells. The length of the bridge between the two daughter cells was 9.141 ± 0.3002 μm in control cells compared with 12.76 ± 0.2603 μm in RPS3-knockdown cells (Fig. 3C).

To further investigate the role of RPS3 in mitosis, progression of mitosis was examined in HeLa-H2B-GFP cells by time-lapse video microscopy. Cells were examined for 12 h at 3 days after control or shRPS3-4 lentivirus infection (Fig. 3D). Half of the shRPS3-4-infected HeLa-H2B-GFP cells entered anaphase and completed cytokinesis, similar to control virus-infected cells, with an average time of 48 min from nuclear envelope breakdown. The other half of the shRPS3-4-infected cells entered metaphase but did not reach anaphase (Fig. 3D, lower panel). Chromosomes aligned, arrested in metaphase, oscillated for several hours, and eventually condensed without segregating (see Supplementary movies S1 and S2).

### RPS3 plays an important role in α-tubulin assembly

**Figure 4:**
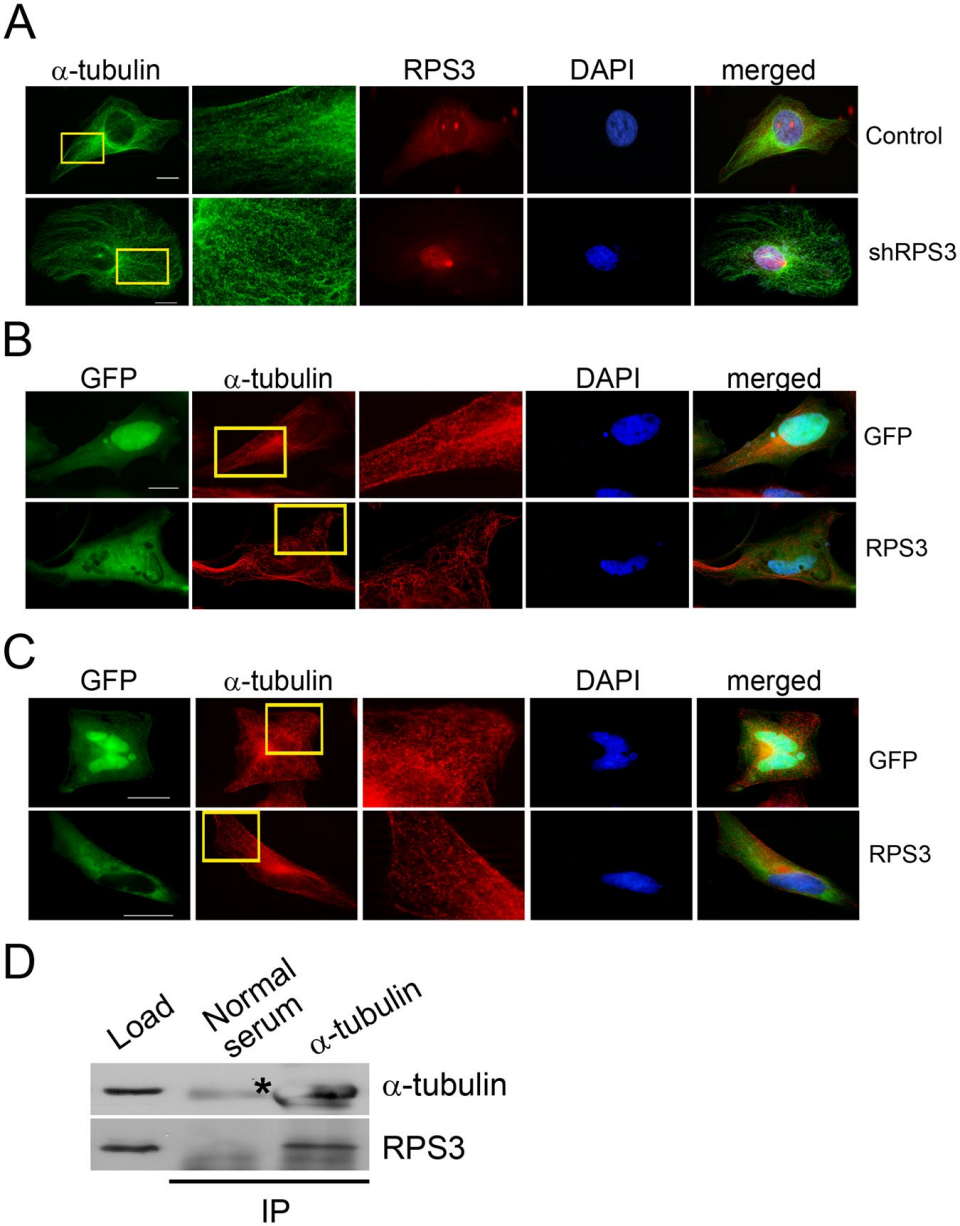
RPS3 interacts with α-tubulin and modulates the organization of α-tubulin. (**A**) Organization of α-tubulin in control and shRPS3-4-transduced HeLa cells. HeLa cells were transduced with the control virus or shRPS3-4 and immunostained with antibodies against RPS3 and α-tubulin. Control, n = 114; shRPS3, n = 134. Experiments were repeated three times. (**B**) α-Tubulin assembly in GFP- or RPS3-GFP-transfected cells. HeLa cells were transfected with an expression construct for GFP or RPS3-GFP and immunostained with an anti-α-tubulin antibody. Control, n = 24; RPS3 = 49. Experiments were repeated two times. (**C**) Expression of RPS3-GFP in RPS3-depleted cells rescues abnormal α-tubulin assembly. HeLa cells were transduced with shRPS3 and transfected with an expression construct for GFP or RPS3-GFP the next day. Cells were further incubated for 48 h and immunostained with an anti-α-tubulin antibody. Control, n = 20; RPS3, n = 51. Experiments were repeated three times. (**D**) RPS3 interacts with α-tubulin in G2/M phase. Cell extracts were prepared from G2/M-arrested HeLa cells, and immunoprecipitation was performed with an anti-α-tubulin antibody. Precipitants were subjected to western blotting with an anti-RPS3 or anti-α-tubulin antibody. *Non-specific band.

Defects in mitotic spindle formation and the α-tubulin network in RPS3-knockdown cells suggest that RPS3 plays an important role in α-tubulin assembly. To examine this possibility, HeLa cells were transduced with shRPS3-4 and assembly of α-tubulin was examined by immunostaining with an antibody against α-tubulin. RPS3 was localized in the cytosol and concentrated in the nucleolus where ribosome assembly occurs in control virus-infected cells. Only a small amount of RPS3 was found in the nucleoplasm of control cells. The amount of RPS3 in the cytosol was greatly reduced and a significant amount of RPS3 was localized in the nucleoplasm as well as the nucleolus of shRPS3-4-transduced cells. α-Tubulin in RPS3-knockdown cells had a dense, bead-like structure, in contrast with the thread-like assembly of microtubules in control cells (Fig. 4A). The dense, bead-like assembly of α-tubulin in RPS3-knockdown cells suggests that RPS3 plays a role in destabilization of microtubule dynamics. To examine this possibility, RPS3-GFP protein was overexpressed in HeLa cells and assembly of α-tubulin was examined by immunostaining with an anti-α-tubulin antibody. Overexpression of RPS3-GFP resulted in a thin, less dense α-tubulin network and severely disoriented assembly of α-tubulin compared with control cells (Fig. 4B). The dense, bead-like assembly of α-tubulin in RPS3-knockdown cells was recovered in cells expressing RPS3-GFP protein, suggesting that RPS3 plays a role in destabilization of microtubule dynamics (Fig. 4C).

The localization of RPS3-GFP differed between control and RPS3-knockdown HeLa cells. In HeLa cells, RPS3-GFP was localized in both the nucleus and cytosol, while it was mostly localized in the cytosol in RPS3-knockdown cells (Fig. 4B,C). In addition to the localization-dependent function of RPS3^14,16^ and tight regulation of the cytosolic RPS3 level^25^, the nuclear localization of overexpressed RPS3-GFP might provide an interesting clue as to the role of RPS3 in the nucleus.

These data suggest that RPS3 interacts with α-tubulin in mitosis and plays a role in formation of the mitotic spindle. To examine this possibility, HeLa cells were arrested in G2/M phase by treatment with nocodazole. α-Tubulin was immunoprecipitated, and co-precipitation of RPS3 was examined by western blotting with an anti-RPS3 antibody. A large amount of α-tubulin protein was precipitated when an anti-α-tubulin antibody was used for immunoprecipitation, while a faint antibody heavy chain band was detected in an immunoprecipitation using normal mouse serum. RPS3 protein co-precipitated with α-tubulin (Fig. 4D), suggesting that RPS3 interacts with α-tubulin during mitosis and plays a role in mitotic spindle formation. In an immunoprecipitation with normal mouse serum, neither α-tubulin nor RPS3 was precipitated (Fig. 4D). In a reverse immunoprecipitation assay with an anti-RPS3 antibody, a small amount of α-tubulin were co-precipitated with RPS3 (see Supplementary Fig. S10B). Cropped blots are shown in Fig. 4D and the full-length blot is presented in Supplementary Fig. S10A.

### Ectopic expression of mCherry-RPS3 rescues the mitotic defects in RPS3-knockdown cells

**Figure 5:**
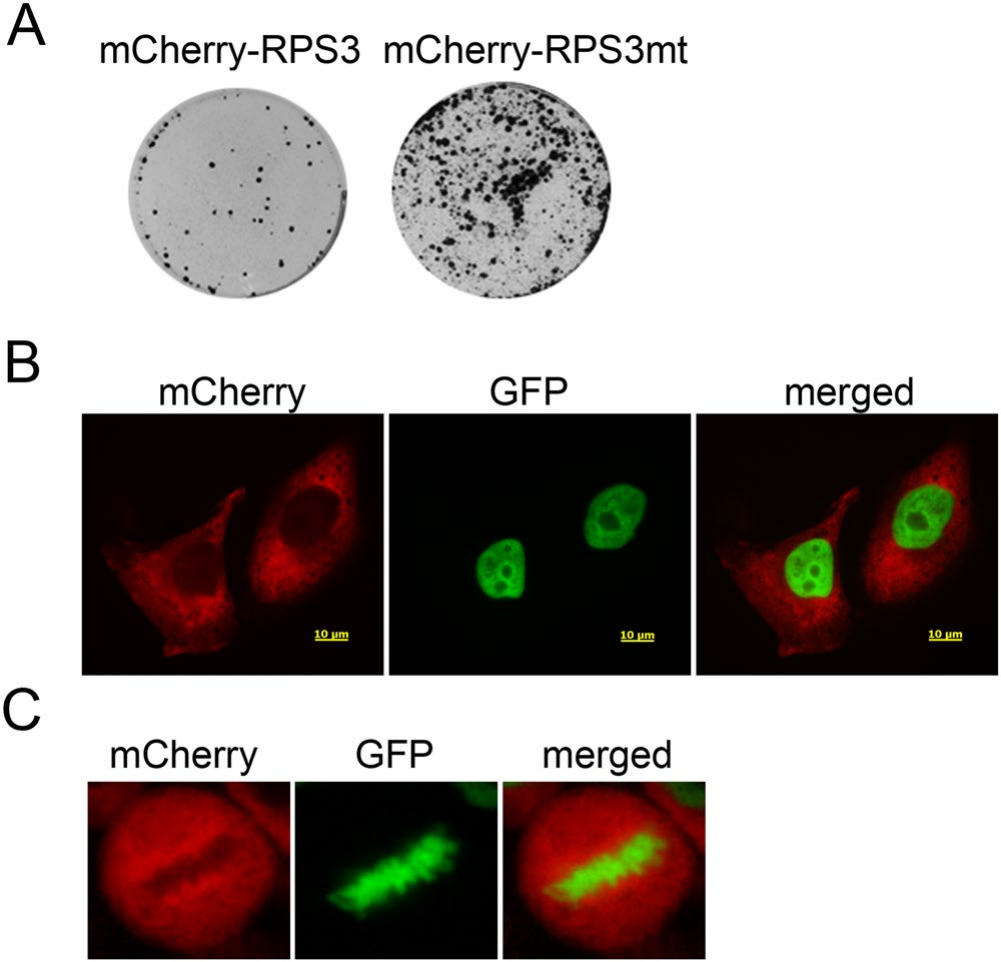
Ectopic expression of mCherry-RPS3mt rescues the mitotic defects. (**A**) Expression of mCherry-RPS3mt rescues mitotic defects in RPS3-knockdown cells. HeLa-H2B-GFP cells were transfected with the pmCherry or pmCherry-*rps3mt* plasmid 48 h after transduction of shRPS3-4 and incubated for a further 7 days in the presence of 2 µg/ml puromycin and 500 µg/ml geneticin. (**B**) mCherry-RPS3mt expression in RPS3-knockdown cells. A low level of mCherry-RPS3 is expressed in the cytosol of RPS3-knockdown cells. (**C**) mCherry-RPS3mt localizes in the mitotic spindle. mCherry-RPS3mt was concentrated around chromosomes and formed mitotic spindle-like threads, and chromosome assembly was not aberrant in metaphase.

To confirm the role of RPS3 in mitosis, mCherry-RPS3 was expressed in RPS3-knockdown cells. A RPS3 mutant that was not suppressed by shRPS3-4 was constructed (RPS3mt) and expressed as a fusion protein with mCherry in HeLa cells transduced with the control virus or shRPS3-4 (see Supplementary Fig. S5). HeLa-H2B-GFP cells were transduced with shRPS3-4 and incubated for 2 days. A plasmid expressing mCherry-RPS3mt or mCherry-RPS3 was transfected, and cells were incubated in the presence of puromycin and neomycin to select both RPS3-knockdown and mCherry-RPS3/mCherry-RPS3mt-expressing cells. Seven days after transfection, a large number of colonies formed with mCherry-RPS3mt-expressing cells, while only a few colonies formed with mCherry-RPS3-expressing cells (Fig. 5A). Cells from a colony were dissociated and cultured, and expression of mCherry-RPS3mt was examined under a fluorescence microscope. A low level of mCherry-RPS3mt was expressed in the cytosol of colony-forming cells (Fig. 5B). mCherry-RPS3mt was concentrated around chromosomes and formed mitotic spindle-like threads in metaphase, and chromosomes did not exhibit aberrant assembly in metaphase, suggesting that ectopic expression of mCherry-RPS3mt rescued the mitotic defects caused by RPS3 knockdown (Fig. 5C).

### Expression of HIV-1 Tat leads to abnormal mitosis and disturbs the localization of RPS3 in the mitotic spindle

**Figure 6:**
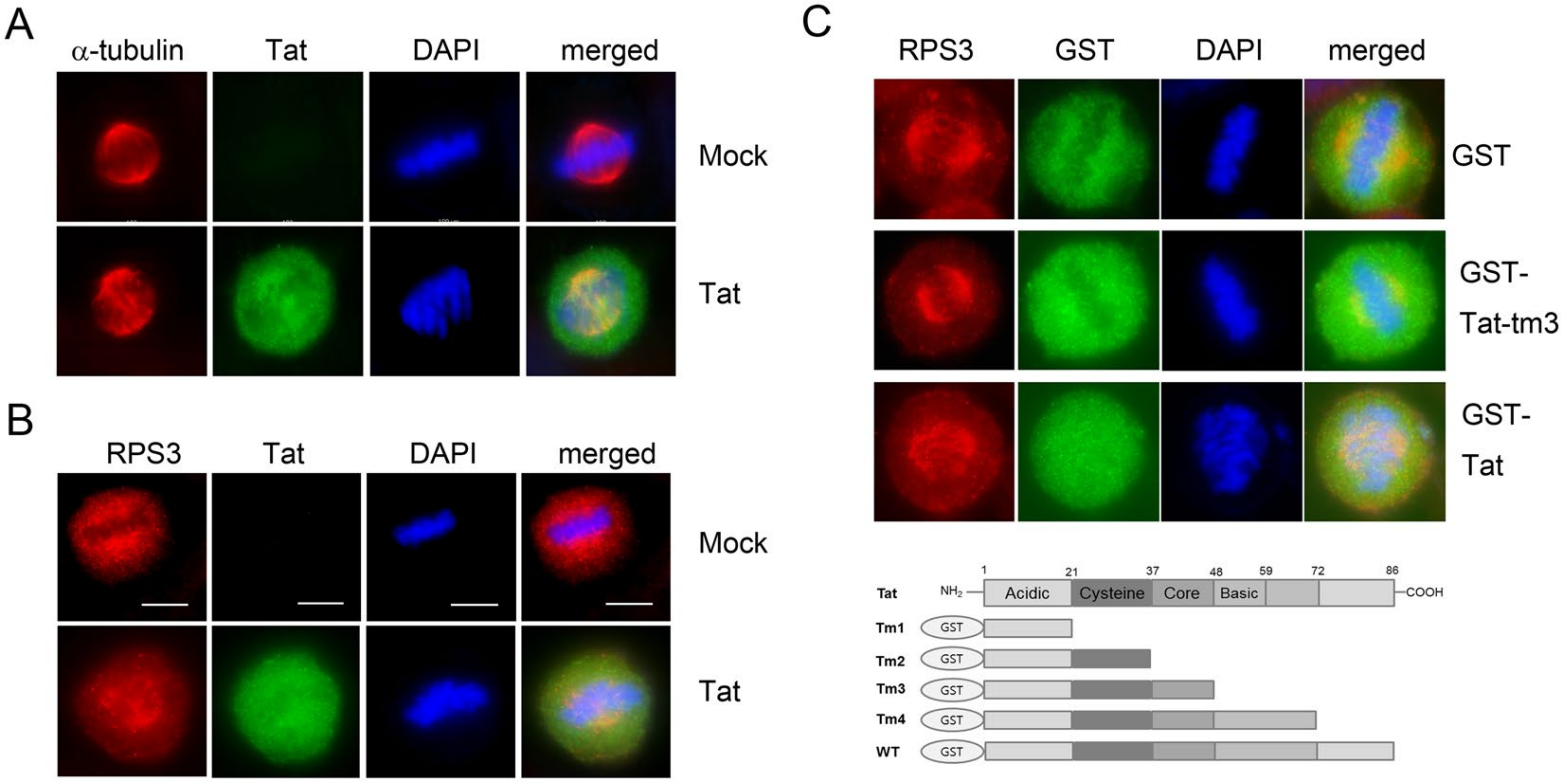
HIV-1 Tat disturbs the mitotic spindle localization of RPS3. (**A**) Mitotic defect in Lenti-Tat-transduced HeLa cells. HeLa cells were transduced with the control virus or Lenti-Tat and stained with an anti-Tat or anti-α-tubulin antibody. Localization of α-tubulin in the mitotic spindle and chromosome segregation were defective in the presence of Tat. Control, n = 60; Tat, n = 139. Experiments were repeated three times. (**B**) HIV-1 Tat disturbs the localization of RPS3 in the mitotic spindle. HeLa cells were transduced with the control virus or Lenti-Tat and stained with anti-Tat and anti-RPS3 antibodies. Control, n = 41; Tat, n = 73. Experiments were repeated two times. (**C**) Disruption of RPS3 localization in the mitotic spindle by HIV-1 Tat requires binding to RPS3 through its basic domain. HeLa cells were transfected with the plasmid expressing GST, GST-Tat, or GST-Tatm3, and the localization of RPS3 in the mitotic spindle was examined by immunostaining with antibodies against RPS3 and GST. GST-Tatm3 lacking the RPS3-binding domain did not inhibit the localization of RPS3 in the mitotic spindle, similar to GST, while wild-type Tat disturbed the localization of RPS3. GST, n = 22; GST-Tatm3, n = 25; GST-Tat, n = 32.

The association of Tat with RPS3 and the role of RPS3 in mitotic spindle formation suggest that Tat is also involved in mitosis. The possible role of Tat in mitosis was examined in HeLa cells transduced with a lentivirus harboring a Tat expression construct (Lenti-Tat) and immunostained with anti-Tat and anti-α-tubulin antibodies. In control virus-infected cells, α-tubulin was concentrated around chromosomes, which showed a typical metaphase arrangement. In the presence of Tat, α-tubulin filaments were longer and denser in metaphase, suggesting that Tat plays a role in mitosis (Fig. 6A).

To examine the effect of Tat on RPS3 in mitosis, Lenti-Tat was transduced into HeLa cells and the localization of RPS3 in mitosis was examined by immunostaining with antibodies against RPS3 and Tat. In control virus-infected cells, RPS3 were concentrated around chromosomes and localized in the mitotic spindle in metaphase. In the presence of Tat, RPS3 was dispersed throughout cells or concentrated around chromosomes, but was aberrantly arranged (Fig. 6B). This result suggests that HIV-1 Tat disturbed the localization of RPS3 in the mitotic spindle or complex formation with α-tubulin during mitosis.

To further examine the role of Tat in RPS3-mediated mitosis, HeLa cells were transfected with a plasmid expressing GST, GST-Tatm3, which does not contain the RPS3-binding domain, or GST-Tat, and the localization of RPS3 in the mitotic spindle was examined by immunostaining with antibodies against RPS3 and GST. Expression of GST-Tat disturbed the localization of RPS3 in the mitotic spindle, resulting in aberrant chromosome assembly in metaphase. Meanwhile, the localization of RPS3 in the mitotic spindle was not inhibited in GST-Tatm3- and GST-expressing cells. This result suggests that HIV-1 Tat associates with RPS3 through its basic domain and disturbs the localization of RPS3 in the mitotic spindle (Fig. 6C).

**Figure 7:**
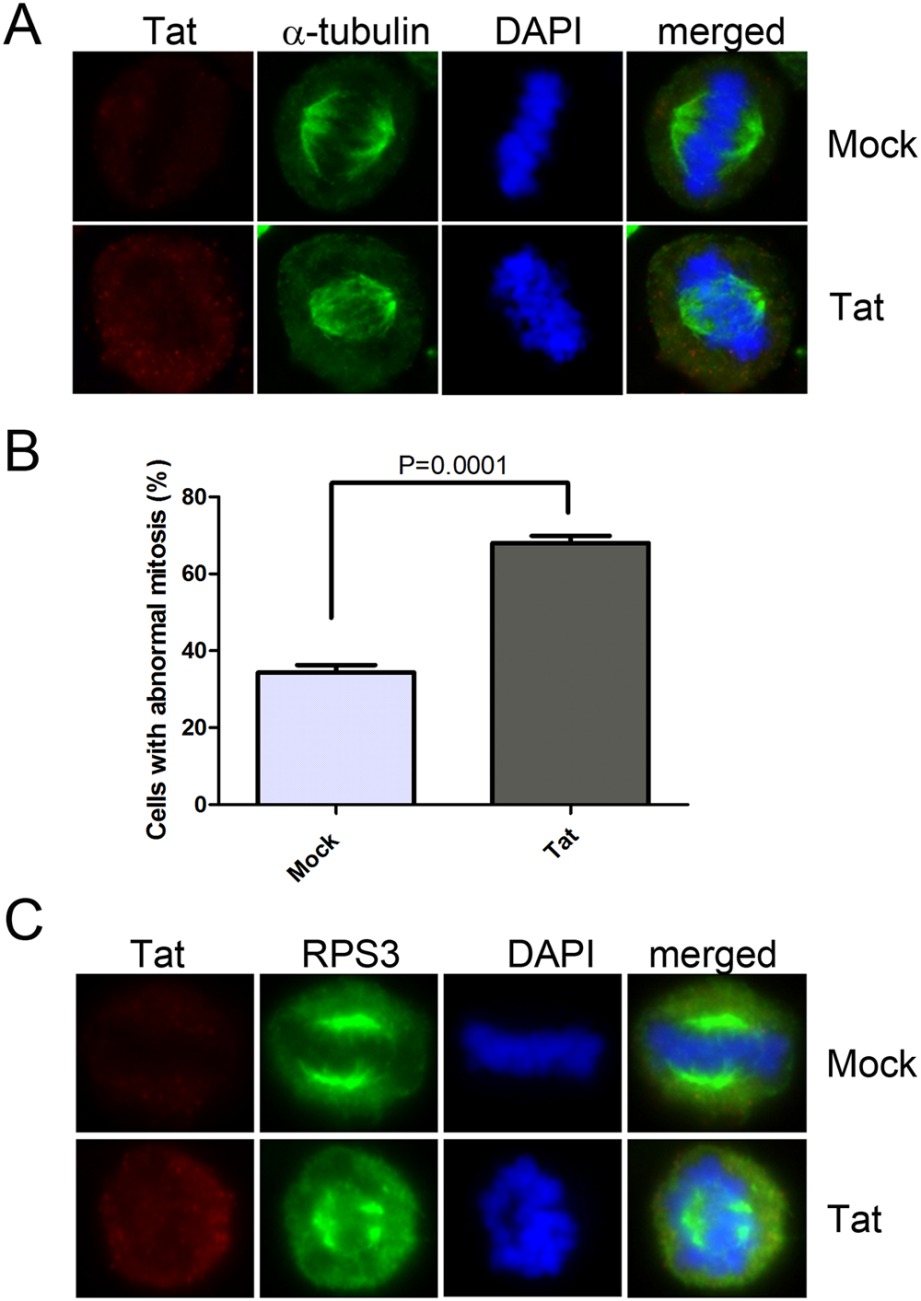
HIV-1 Tat disturbs mitotic spindle formation in Jurkat cells. (**A**) A representative figure of the mitotic spindle defect in Lenti-Tat-transduced Jurkat cells. Jurkat cells were transduced with the control virus or Lenti-Tat and immunostained with antibodies against Tat and α-tubulin 3 days after infection. Control, n = 189; Tat, n = 220 from three independent experiments. (**B**) Mitotic defects in Jurkat cells. The percentage of cells showing mitotic spindle formation defects and misaligned chromosomes is plotted. Data are presented as mean ± SEM from three independent experiments. Statistical analysis was performed by an unpaired *t* test. Control, n = 189; Tat, n = 220; P < 0.0001. (**C**) Localization of RPS3 in the mitotic spindle in the presence of Tat. Lenti-Tat-infected Jurkat cells were immunostained with antibodies against Tat and RPS3 at 3 days after infection. RPS3 was displaced from the mitotic spindle pole, and an aberrant spindle was formed. Control, n = 44; Tat, n = 132. Experiments were repeated two times.

Mitotic defects in the presence of Tat were also examined in Jurkat cells. Aberrant mitotic spindle formation and misaligned chromosomes in metaphase were also significantly increased in Lenti-Tat-infected Jurkat cells (Fig. 7A,B). The mitotic spindle was mis-oriented and fragmented (Fig. 7A). Moreover, RPS3 was displaced from the mitotic spindle pole where the majority of RPS3 protein localized in mock virus-infected Jurkat cells (Fig. 7C), consistent with the results in HeLa cells. These data suggest that Tat induces mitotic defects through an interaction with RPS3 in Jurkat cells as well as HeLa cells.

## Discussion

HIV-1 Tat regulates host cell proliferation through various mechanisms including apoptosis^26^, translation^27,28^, and signal transduction^29–31^ as well as mitosis^10^. Here, we provide new evidence that Tat may modulate cell proliferation by inhibiting mitosis through an interaction with RPS3. We also provide evidence that RPS3 plays a role in mitosis. This study showed that RPS3 protein localized in the mitotic spindle in mitosis (Fig. 2) and depletion of RPS3 resulted in aberrant mitotic spindle formation, segregation failure, and defective abscission (Fig. 3), suggesting an important function of RPS3 in mitosis. The interaction of RPS3 with α-tubulin in mitosis (Fig. 4D) as well as the disruption of α-tubulin dynamics in RPS3-depleted or -overexpressing cells (Fig. 4A–C) demonstrates that RPS3 interacts with microtubules and modulates microtubule dynamics.

Recent studies showed that RPS3 is phosphorylated by various cellular kinases and translocates into nucleus, where it plays a role in DNA repair and apoptosis^12,16,32^. In addition, RPS3 is also involved microbial pathogenesis^18^. The level of nuclear RPS3 was increased in the presence of Tat (Fig. 1). Given that nuclear RPS3 functions in DNA repair and regulation of gene expression, regulation of the level of nuclear RPS3 by Tat might provide an interesting insight into the interaction of HIV-1 with host cells. However, it is unknown how RPS3 accumulates in the nucleus in the presence of Tat. One possibility is that nuclear import of RPS3 is increased by its association with Tat. RPS3 undergoes conventional nuclear import via an interaction with importin α^18^. Controversially, Tat is reported to undergo nuclear import independently of the importin α/β heterodimer, although it contains a nuclear localization signal at its N-terminus^33,34^. RPS3 in Tat-expressing cells may be imported into the nucleus as a complex with Tat, as well as in the conventional importin-dependent manner, leading to an increased level of nuclear RPS3. Alternatively, Tat may indirectly induce nuclear accumulation of RPS3 by promoting its phosphorylation. Diverse protein kinases including PKCδ, Akt, and Erk1/2 must phosphorylate RPS3 prior to its nuclear translocation^14,16,32^. Tat activates various protein kinases including PKC, Akt, and Erk1/2^30,35,36^. Activation of Akt and Erk1/2 by Tat may induce phosphorylation of RPS3, resulting in its nuclear accumulation. Further studies are required to examine how expression of Tat induces nuclear accumulation of RPS3.

In immunoprecipitation assays with anti-Tat and anti-RPS3 antibodies, RPS6 and RPL26 were co-precipitated with RPS3 and Tat (Supplementary Fig. S1). This might be due to the interaction of Tat with RPS3 incorporated into the ribosome complex, where most cytosolic RPS3 is located^37^. RPS3 may mediate the association of RPS6 and RPL26 with Tat. Alternatively, RPS6 or RPL26 might directly interact with Tat and mediate its association with RPS3. However, the nuclear level of RPS3 was increased in the presence of Tat (Fig. 1D), while the nuclear localizations of RPS6 and RPL26 were not (Supplementary Fig. S2). Moreover, RPS6 did not localize to the mitotic spindle pole. By contrast, RPS3 was localized in the mitotic spindle, and this was disrupted in the presence of Tat (Supplementary Fig. S4C). These results suggest that the increased nuclear level of RPS3 and disruption of the localization of RPS3 in the mitotic spindle were due to a direct interaction between RPS3 and Tat. Further studies are required to elucidate the relationship between RPS3, RPL26, RPS6 and Tat.

Jang *et al*. also reported that RPS3 and microtubules interact, that RPS3 localizes to the mitotic spindle, and that RPS3-knockdown cells exhibit mitotic spindle defects^21^. However, the authors suggested that RPS3 is required for microtubule polymerization. They showed that depletion of RPS3 decreased the density of the mitotic spindle and slowed microtubule polymerization. Here, we demonstrated that depletion of RPS3 led to assembly of a dense and disorganized α-tubulin network, while overexpression of RPS3 resulted in formation of a thin and less dense α-tubulin network and severely disoriented α-tubulin assembly (Fig. 4A,B). This dense and disorganized α-tubulin assembly was rescued by ectopic expression of GFP-RPS3 (Fig. 4C), suggesting that RPS3 plays a role in microtubule destabilization. The discrepancy between these two studies might be due to the different assays used to investigate the role of RPS3 in microtubule assembly. Jang *et al*. measured microtubule repolymerization after removal of the mitotic spindle from mitotic cells, while we examined microtubule assembly in interphase cells following knockdown or overexpression of RPS3. Further studies are required to elucidate the role of RPS3 in microtubule dynamics.

RPS3 interacted with α-tubulin in G2/M phase cells, and depletion of RPS3 led to dense and disorganized α-tubulin assembly (Fig. 4). Depletion of RPS3 also resulted in aberrant mitotic spindle formation, segregation failure, and defective abscission (Fig. 3). Aberrant mitotic spindle formation was also observed in Tat-expressing cells (Figs 6 and 7). Chromosome number aberrations caused by expression of Tat in *Drosophila* larvae embryos via an interaction with microtubules^10^ and the results we present here suggest that Tat interacts with RPS3 and disturbs the interaction of RPS3 with α-tubulin, resulting in mitotic defects in Tat-expressing cells. A significant amount of RPS3 protein in metaphase was dispersed throughout cells and the localization of RPS3 around chromosomes was disturbed in the presence of Tat (Figs 6B and 7C), demonstrating that Tat causes defects in the interaction between RPS3 and α-tubulin. Further studies are required to examine the relationship between Tat, RPS3, and tubulin during mitosis.

Treatment with tubacin, a specific inhibitor of HDAC6, increased the amount of Tat associated with RPS3 in a dose-dependent manner (Fig. 1C). This suggests that acetylation of Tat at K28 interferes with the involvement of RPS3 in mitotic spindle formation through an increase in the interaction between Tat and RPS3, resulting in microtubule assembly defects in cell cycle. Taken together, our results suggest that Tat could make a perturbation in microtubule assembly^9,22^ in cells that were infected by HIV-1 or absorbed Tat protein secreted from infected cells.

Enhanced apoptosis has been reported in lymphocytes of HIV-1-infected individuals^38–40^. HIV-1 Tat induces apoptosis of lymphocytes by diverse mechanisms including modulation of Bax and Bcl-2^41^, disruption of the mitochondrial membrane potential, and a subsequent increase in reactive oxygen species^42^ or upregulation of CD95L^43^. The data demonstrating the role of HIV-1 Tat in mitosis through an interaction with RPS3 in Jurkat cells (Fig. 7) suggest that Tat induces apoptosis in HIV-1-infected lymphocytes. Given that mitotic catastrophe induces apoptosis^44^, the data we present here might provide new insight into HIV-1 Tat-mediated apoptosis in HIV-1-infected individuals and the consequent reduction of lymphocytes in AIDS patients.

Taken together, these data suggest that Tat interacts with RPS3 and disturbs the localization of RPS3 around chromosomes, suggesting that Tat inhibits mitosis via an interaction with RPS3.

## Materials and Methods

### Cells and reagents

HeLa cells and 293FT human epithelial kidney cells were cultured at 37 °C, 5% CO_2_ in Dulbecco’s modified Eagle’s medium (DMEM; Hyclone Laboratories Inc.) supplemented with 10% fetal bovine serum (FBS; Gibco-BRL, Invitrogen,) and penicillin-streptomycin (Gibco-BRL, Invitrogen). HeLa cells stably expressing H2B–GFP (HeLa-H2B-GFP) were kindly provided by Dr. Toru Hirota (Japanese Foundation for Cancer Research, Tokyo) and cultured in DMEM supplemented with 10% FBS and penicillin-streptomycin at 37 °C in a 5% CO_2_ incubator. Jurkat cells were cultured at 37 °C, 5% CO_2_ in RPMI 1640 medium (Hyclone Laboratories Inc.) supplemented with 2 mM glutamine, 10% FBS, and penicillin-streptomycin.

### Plasmids

The *mcherry* sequence was amplified from pH2B-*mcherry* using the following two primers containing *Age*I or *Bsp*EI at their N- or C-terminus, respectively: mc1; 5′-GCTACCGGTATGGTGAGCAAGGG-3′ and mc2; 5′-CTCTCCGGACTTGTACAGCTCATCCATG-3′. The PCR product was digested with *Age*I and *Bsp*EI and inserted into pEYFP-C1 (Clontech) that had been digested with these enzymes (pmCherry-c1). RPS3 was amplified by PCR from pEGFP-C1-*rps3* (a kind gift from Dr. Ahn^16^) using the following primers: rps3-1; 5′-CTCAGATCTATGGCAGTGCAAATATCCAAG-3′ and rps3-2; 5′-GTGAAGCTTTTATGCTGTGGGGACTGGC-3′. The amplification conditions were 95 °C for 1 min, followed by 25 cycles of denaturation at 95 °C for 1 min, annealing at 58 °C for 30 s, and elongation at 72 °C for 30 s. Amplified DNA was digested with *Bgl*II and *Hind*III and ligated into pmCherry-c1 that had been digested with these enzymes. Mutant *rps3* was generated by PCR amplification of pEGFP-C1-*rps3* with the mutant primers. First, two independent PCRs were performed with the following two sets of primers: Set1; rps3-1 plus rps3-6; 5′-GTGGTGGGCAGTATCTCATCCTTAGGTTCCACAATGCTCACGTG-3′, and Set2; rps3-5; 5′-CACGTGAGCATTGTGGAACCTAAGGATGAGATACTGCCCACCAC-3′ plus rps3-2. PFU (BIONEER) was used as the DNA polymerase, and the PCR conditions were the same as those used for wild-type *rps3* amplification. The PCR products were run on a 1% agarose gel, the DNA bands were excised, and both gel fragments were put into a 1.5 ml microtube containing 100 μl of DW. The gel slices were then incubated for 15 min at 37 °C. Thereafter, 20 μl of DNA solution was removed from each tube, mixed, and used as a template for the second PCR. In the second PCR, rps3-1 and rps3-2 were used as the primer set. The second PCR products were run on a 0.9% agarose gel and purified using the MinElute Gel Extraction kit (QIAGEN). Purified DNA was digested with *Bgl*II and *Hind*III and ligated into pmCherry-C1 that had been digested with these enzymes (pmCherry-*rps3mt*). Amplified DNA was verified by sequencing after subcloning.

Constructs harboring wild-type Tat and Tat deletion mutants (pGEX4T*-tatTm*, pGEX4T*-tatTm2*, pGEX4T*-tatTm3*, pGEX4T-*tatTm4* and pGEX4T*-tatTm5*) were digested with *Bam*HI and *Not*I. The DNA fragments were ligated into the pEBG plasmid, which had been digested with the same restriction enzymes, to generate pEBG-*tatTm1*, pEBG*-tatTm2*, pEBG*-tatTm3*, pEBG*-tatTm4* and pEBG-*tatTm5*.

### Antibodies

The anti-RPS3 antibody used for western blotting was purchased from Adipogen (cat. no. AG-25A-0077). A polyclonal antibody against the synthetic peptide corresponding to the sequence of human RPS3 (Cell Signaling, cat. no. 2579) was used for immunostaining. An antibody against amino acids 1–50 of human RPS3 was purchased from Abcam (cat. no. ab140676) and used for immunoprecipitation. An anti-Tat antibody was produced from hybridoma cells (1D9, cat. no. 7373) that were kindly provided by the NIH AIDS Research and Reference Reagent program. Anti-α-tubulin (B-7, cat. no. SC-5286), anti-RPS6 (cat. no. sc-5286), and anti-PARP (cat. no. sc-7150) antibodies were purchased from Santa Cruz. An anti-RPL26 antibody (cat. no. ab59567) was purchased from Abcam. An anti-α-tubulin (cat. no. 600-401-880) was purchased from Rockland Inc. Fluorochrome-conjugated antibodies were purchased from Jackson Immunoresearch Laboratories Inc.

### Transfection

HEK 293FT cells were plated on a 12- or 6-well plate at a density of 3 × 10^5^ or 6 × 10^5^ cells/well, respectively, and incubated for 24 h. Thereafter, cells were transfected with 0.7 µg (12-well plate) or 1.0 µg (6-well plate) of plasmid DNA using Lipofectamine (cat. no. 18324-020) and Plus reagents (cat. no. 11514-015) (Thermo-Fisher).

HeLa-H2B-GFP cells were plated on a 6-well plate at a density of 3 × 10^5^ cells/well the day after shRPS3-4 transduction, incubated for 24 h, and transfected with 1 μg of pmCherry-rps3 or pmCherry-rps3mt using Lipofectamine and Plus reagents.

### Immunoprecipitation

HEK 293FT cells were transfected with the Tat expression construct and incubated for 16 h. Harvested cells were resuspended in buffer A (10 mM HEPES pH 7.5, 1.5 mM MgCl_2_, 10 mM KCl, 0.5 mM DTT, and 0.05% NP-40) and incubated for 20 min at 4 °C with rotation. Cells were centrifuged at 3,000 rpm at 4 °C for 5 min, and the supernatant was designated the cytosolic fraction. Pellets were resuspended in buffer A, briefly sonicated, and centrifuged at 12,000 rpm at 4 °C for 15 min. The supernatant was designated the nucleoplasmic fraction and the precipitant was designated the nucleolar fraction. The protein concentration in the cytosolic fraction was quantified by the BCA assay, and 500 µg of this fraction was subjected to immunoprecipitation with an anti-RPS3 antibody. The same volume of the nucleoplasmic fraction was subjected to immunoprecipitation with this antibody. HeLa cells were treated with 100 ng/ml nocodazole and incubated for 16 h. Cells were resuspended in PBS containing 1% NP-40 and protease inhibitors and incubated for 30 min at 4 °C with rotation. The supernatant was separated by centrifugation at 12,000 rpm, 4 °C for 30 min. An anti-RPS3 or anti-α-tubulin antibody was added to 500 μg of the cell extract and incubated for 1 h on ice. Thereafter, 10 μl of protein G-Sepharose CL4B (Amersham Pharmacia Biosciences) was added and incubated overnight at 4 °C with rotation. Protein G beads were pelleted by centrifugation at 1,000 rpm, 4 °C for 5 min and washed three times with PBS containing 1% NP-40. Bound proteins were eluted by boiling with SDS-loading dye and separated by SDS-PAGE, followed by western blotting with an anti-Tat, anti-RPS3, or anti-α-tubulin antibody.

### Western blotting

Samples were subjected to SDS-PAGE (10% for PARP, α-tubulin, and RPS3; 15% for RPS6, RPL26, and Tat) and transferred to polyvinylidene difluoride membrane (BIO-RAD) by electroblotting. The protein concentration of the cytosolic fraction was quantified by the BCA assay and 20 μg of this fraction was immunoblotted. The same volume of the total and nuclear fractions was subjected to western blotting. Anti-RPS3 (1:1000 dilution), anti-Tat (1:1000 dilution), anti-α-tubulin (1:5000 dilution), anti-RPL26 (1:1000 dilution), anti-PARP (1:1000), and anti-RPS6 (1:1000 dilution) antibodies were used. The secondary antibodies were goat anti-mouse (172–1011 BIO-RAD) and donkey anti-rabbit (cat. no. NA934, Amersham Pharmacia Biosciences) conjugated to horseradish peroxidase. Chemiluminescent signals were detected using ECL western blot detection reagents (GE Healthcare).

### Immunofluorescence

HeLa cells were seeded onto circular glass coverslips (18 mm, Marienfeld, Lauda-Königshofen, Germany) in a 12-well plate and incubated for 24 h. Cells were transfected with 0.7 μg of the expression construct encoding Tat or transduced with shRPS3-expressing lentivirus using 8 μg/ml polybrene (SIGMA 107689, USA). After 2 days, the cells were fixed for 10 min with 4% paraformaldehyde, permeabilized with 0.1% Triton X-100 for 5 min, and blocked with 4% bovine serum albumin for 1 h. To examine the role of RPS3 in the mitotic spindle, cells were stained with anti-RPS3 (1:25 dilution) and α-tubulin (B-7, 1:200 dilution) antibodies followed by a FITC-conjugated anti-mouse antibody (1:200 dilution) and a rhodamine-conjugated anti-rabbit antibody (1:200 dilution), respectively. Cells were immunostained with anti-Tat (1:200 dilution) and anti-RPS3 antibodies followed by a FITC-conjugated anti-mouse antibody and a rhodamine-conjugated anti-rabbit antibody, respectively, to examine the localization of RPS3 at the mitotic spindle. Cells were co-stained with anti-Tat and anti-α-tubulin (cat. no. 600-401-880) antibodies followed by a FITC-conjugated anti-mouse antibody and a rhodamine-conjugated anti-rabbit antibody, respectively, to examine the role of Tat at the mitotic spindle. HEK 293FT cells were transfected with the control or Tat expression construct, incubated for 24 h, and stained with anti-Tat and anti-RPS3 antibodies followed by a FITC-conjugated anti-mouse antibody (1:200 dilution) and a rhodamine-conjugated anti-rabbit antibody, respectively. Jurkat cells were transduced with the control or Tat-expressing lentivirus using 8 μg/ml polybrene (SIGMA 107689) and then stained with anti-Tat and anti-RPS3 antibodies or anti-Tat and anti-α-tubulin (cat. no. 600-401-880) antibodies followed by Cy3-conjugated anti-mouse and Alexa Fluor 488-conjugated anti-rabbit antibodies. Cells were visualized using an inverted phase fluorescence microscope (Carl Zeiss, Axiovert 200 with Axiovision software, Leica, DMi8 with LAS X software). The length of the bridge between two daughter cells was measured using the distance tool of LAS X software.

### Lentivirus production and concentration

Lentiviruses were produced as described previously with some modifications^45^. shRNA targeting human RPS3 was purchased from SIGMA (SIGMA Mission shRNA, SHCLNG-NM_001005). HEK 293FT^46^ cells were seeded in a 6-well plate 24 h prior to transfection. HEK 293FT cells were transfected with the transfer vector, the packaging plasmid (psPax2, 0.48 µg each), and 0.24 μg of the envelope plasmid (pMD2.G)^47^ using Lipofectamine Reagent (Invitrogen) and incubated for 48 h. The supernatant was harvested, cleared by centrifugation at 1,500 rpm for 5 min, and filtered through a 0.22 μm cellulose acetate filter (Sartorius Stedim Biotech). Lenti-Tat was produced using the same procedure, except that pLenti-*Tat* was used as the transfer vector. HEK 293FT cells were transduced with Lenti-Tat and expression of Tat was analyzed by immunoblotting with an anti-Tat antibody.

### Live cell imaging

HeLa-H2B-GFP cells were transduced with the control or shRPS3-4 virus and incubated for 2 days. Cells were plated onto a chamber slide (Lab-Tek cat.no. 155409) and incubated for a further 24 h. Images were acquired using a 40×, NA 0.6 dry objective lens every 5 min for 24 h in a chamber at 37 °C in 5% CO_2_. GFP fluorescence was imaged using a conventional laser excitation and filter set. Images were processed using ImageJ software.

### Colony formation assay

HeLa-H2B-GFP cells were transduced with shRPS3-4. Cells were transfected with the pmCherry-rps3 or pmCherry-rps3mt plasmid and incubated in DMEM containing 2 μg/ml puromycin and 500 μg/ml neomycin. After 1 week, colonies were stained with crystal violet.

### Subcellular fractionation

Cells transfected with the Tat expression construct were harvested, resuspended in buffer A (10 mM HEPES pH 7.5, 1.5 mM MgCl_2_, 10 mM KCl, 0.5 mM DTT, and 0.05% NP-40), and incubated for 20 min at 4 °C with rotation. Cells were centrifuged at 3,000 rpm, 4 °C for 5 min, and the supernatant was designated the cytoplasmic fraction and the precipitant was designated the nuclear fraction.

### GST pulldown assay

293FT cells were transfected with plasmids expressing GST-fused Tat deletion mutants (pEBG-*tatTm1*, pEBG*-tatTm2*, pEBG*-tatTm3*, pEBG*-tatTm4* and pEBG-*tatTm5*), incubated for 48 h, and resuspended in PBS supplemented with 1% NP-40 and protease inhibitors. Thereafter, 10 µl of glutathione agarose 4B beads (cat. no. 745500.10; Macherey-Nagel) was added to the cell extract and incubated for 3 h. Beads were washed three times with PBS supplemented with 1% NP-40 and 250 mM NaCl. Bound proteins were eluted by boiling in SDS-PAGE buffer and analyzed by 10% SDS-PAGE followed by western blotting with an anti-RPS3 antibody. Blots were stripped and re-probed with an anti-GST antibody.

### Matrix-assisted laser desorption time-of-flight mass spectrometry

Wild-type Tat fused to maltose-binding protein (MBP) was bound to amylose resin (cat no. E8021S; NEB). As a control, mutant Tat (mTat), which is deficient in transactivation due to replacement of lysine 41 by glutamate (TatK41E), was fused to MBP and bound to amylose resin. HEK 293FT cells were harvested from five 100 mm dishes and resuspended in buffer B (50 mM Tris-Cl, pH 8.0, and 0.5% NP-40) containing 100 mM NaCl and precleared by passing thrice through 3 ml of mTat-MBP-amylose. The flow-through was passed through 200 μl of Tat-MBP-amylose or mTat-MBP-amylose, and the resin was washed with buffer B containing 200 mM NaCl. Bound proteins were eluted stepwise with buffer B containing 300 mM, 400 mM, 500 mM, or 1 M NaCl. Proteins that bound only to wild-type Tat were analyzed by matrix-assisted laser desorption time-of-flight mass spectrometry (Yonsei Proteome Research Center, South Korea).

### Effects of acetylation of Tat at K28 on its association with RPS3

293FT cells were transfected with the Tat expression construct and incubated for 24 h in the presence of 0, 1, or 3 μM tubacin, an HDAC6 inhibitor. Cell extracts were prepared by resuspending cells in lysis buffer containing NP-40 and the protein concentration was determined by the BCA assay. Thereafter, 500 μg of each cell extract was subjected to immunoprecipitation with an anti-RPS3 antibody. Bound proteins were analyzed by immunoblotting with anti-Tat, anti-α-tubulin, anti-GAPDH, and anti-RPS3 antibodies.

### Statistical analyses

The Student’s *t* test was used for most statistical analyses. A one-way ANOVA was used to analyze data acquired in the MTT assay. Prism 5 statistical software was used for the analyses (GraphPad Software). *P* values that were <0.05 were considered to be statistically significant.

### Data availability

The datasets generated and/or analyzed in the current study are available from the corresponding author upon reasonable request.

## Electronic supplementary material

Supplementary information

## References

[1] Huigen MC, Kamp W, Nottet HS (2004). Multiple effects of HIV-1 trans-activator protein on the pathogenesis of HIV-1 infection. Eur J Clin Invest.

[2] SenGupta DN, Berkhout B, Gatignol A, Zhou AM, Silverman RH (1990). Direct evidence for translational regulation by leader RNA and Tat protein of human immunodeficiency virus type 1. Proc Natl Acad Sci USA.

[3] Braddock M (1990). A nuclear translational block imposed by the HIV-1 U3 region is relieved by the Tat-TAR interaction. Cell.

[4] Albini A (1996). The angiogenesis induced by HIV-1 tat protein is mediated by the Flk-1/KDR receptor on vascular endothelial cells. Nat Med.

[5] Lotz M, Clark-Lewis I, Ganu V (1994). HIV-1 transactivator protein Tat induces proliferation and TGF beta expression in human articular chondrocytes. J Cell Biol.

[6] Milani D (1996). Extracellular human immunodeficiency virus type-1 Tat protein activates phosphatidylinositol 3-kinase in PC12 neuronal cells. J Biol Chem.

[7] Wu Z, Cavallaro U, Marchisio PC, Soria MR, Maier JA (1998). Fibronectin modulates the effects of HIV-1 Tat on the growth of murine Kaposi’s sarcoma-like cells through the down-regulation of tyrosine phosphorylation. Am J Pathol.

[8] Bethel-Brown C (2011). HIV-1 Tat-mediated induction of platelet-derived growth factor in astrocytes: role of early growth response gene 1. J Immunol.

[9] Chen D, Wang M, Zhou S, Zhou Q (2002). HIV-1 Tat targets microtubules to induce apoptosis, a process promoted by the pro-apoptotic Bcl-2 relative Bim. EMBO J.

[10] Battaglia PA (2005). The HIV-Tat protein induces chromosome number aberrations by affecting mitosis. Cell Motil Cytoskeleton.

[11] Liu M (2014). Modulation of Eg5 activity contributes to mitotic spindle checkpoint activation and Tat-mediated apoptosis in CD4-positive T-lymphocytes. J Pathol.

[12] Wilson DM, Deutsch WA, Kelley MR (1993). Cloning of the Drosophila ribosomal protein S3: another multifunctional ribosomal protein with AP endonuclease DNA repair activity. Nucleic Acids Res.

[13] Yacoub A, Augeri L, Kelley MR, Doetsch PW, Deutsch WA (1996). A Drosophila ribosomal protein contains 8-oxoguanine and abasic site DNA repair activities. EMBO J.

[14] Kim TS, Kim HD, Kim J (2009). PKCdelta-dependent functional switch of rpS3 between translation and DNA repair. Biochim Biophys Acta.

[15] Kim, H. D., Lee, J. Y. & Kim, J. Erk phosphorylates threonine 42 residue of ribosomal protein S3. *Biochem Biophys Res Commun* **333**, 110–115, S0006-291X(05)01069-7 (2005). 10.1016/j.bbrc.2005.05.079 15950189

[16] Lee SB (2010). Ribosomal protein S3, a new substrate of Akt, serves as a signal mediator between neuronal apoptosis and DNA repair. The Journal of biological chemistry.

[17] Jang CY, Lee JY, Kim J (2004). RpS3, a DNA repair endonuclease and ribosomal protein, is involved in apoptosis. FEBS Lett.

[18] Wan F (2011). IKKbeta phosphorylation regulates RPS3 nuclear translocation and NF-kappaB function during infection with Escherichia coli strain O157:H7. Nat Immunol.

[19] Tian Y (2015). RPS3 regulates melanoma cell growth and apoptosis by targeting Cyto C/Ca2+/MICU1 dependent mitochondrial signaling. Oncotarget.

[20] Nagao-Kitamoto H (2015). Ribosomal protein S3 regulates GLI2-mediated osteosarcoma invasion. Cancer Lett.

[21] Jang CY, Kim HD, Zhang X, Chang JS, Kim J (2012). Ribosomal protein S3 localizes on the mitotic spindle and functions as a microtubule associated protein in mitosis. Biochem Biophys Res Commun.

[22] Huo L (2011). Tat acetylation regulates its actions on microtubule dynamics and apoptosis in T lymphocytes. J Pathol.

[23] Huo L (2011). Regulation of Tat acetylation and transactivation activity by the microtubule-associated deacetylase HDAC6. The Journal of biological chemistry.

[24] Choi JR, Lee SY, Shin KS, Choi CY, Kang SJ (2017). p300-mediated acetylation increased the protein stability of HIPK2 and enhanced its tumor suppressor function. Sci Rep.

[25] Kim HD (2010). RpS3 translation is repressed by interaction with its own mRNA. J Cell Biochem.

[26] Che X (2014). HIV-1 Tat-mediated apoptosis in human blood-retinal barrier-associated cells. PLoS One.

[27] Braddock M, Powell R, Blanchard AD, Kingsman AJ, Kingsman SM (1993). HIV-1 TAR RNA-binding proteins control TAT activation of translation in Xenopus oocytes. FASEB J.

[28] Cai R, Carpick B, Chun RF, Jeang KT, Williams BR (2000). HIV-I TAT inhibits PKR activity by both RNA-dependent and RNA-independent mechanisms. Arch Biochem Biophys.

[29] Barillari G (1999). The Tat protein of human immunodeficiency virus type-1 promotes vascular cell growth and locomotion by engaging the alpha5beta1 and alphavbeta3 integrins and by mobilizing sequestered basic fibroblast growth factor. Blood.

[30] Deregibus MC (2002). HIV-1-Tat protein activates phosphatidylinositol 3-kinase/ AKT-dependent survival pathways in Kaposi’s sarcoma cells. The Journal of biological chemistry.

[31] Conaldi, P. G. *et al*. Human immunodeficiency virus-1 tat induces hyperproliferation and dysregulation of renal glomerular epithelial cells. *Am J Pathol* **161**, 53–61, S0002-9440(10)64156-9 (2002). 10.1016/S0002-9440(10)64156-9 PMC1850697 12107089

[32] Yadavilli S, Hegde V, Deutsch WA (2007). Translocation of human ribosomal protein S3 to sites of DNA damage is dependant on ERK-mediated phosphorylation following genotoxic stress. DNA Repair (Amst).

[33] Fulcher AJ, Jans DA (2003). The HIV-1 Tat transactivator protein: a therapeutic target?. IUBMB Life.

[34] Efthymiadis A, Briggs LJ, Jans DA (1998). The HIV-1 Tat nuclear localization sequence confers novel nuclear import properties. The Journal of biological chemistry.

[35] Borgatti P (1997). Extracellular HIV-1 Tat protein activates phosphatidylinositol 3- and Akt/PKB kinases in CD4+ T lymphoblastoid Jurkat cells. Eur J Immunol.

[36] Mischiati C (1999). Extracellular HIV-1 Tat protein differentially activates the JNK and ERK/MAPK pathways in CD4 T cells. AIDS.

[37] Kim TS (2006). Interaction of Hsp90 with ribosomal proteins protects from ubiquitination and proteasome-dependent degradation. Mol Biol Cell.

[38] Meyaard L (1992). Programmed death of T cells in HIV-1 infection. Science.

[39] Groux H (1992). Activation-induced death by apoptosis in CD4+ T cells from human immunodeficiency virus-infected asymptomatic individuals. J Exp Med.

[40] Terai C, Kornbluth RS, Pauza CD, Richman DD, Carson DA (1991). Apoptosis as a mechanism of cell death in cultured T lymphoblasts acutely infected with HIV-1. The Journal of clinical investigation.

[41] Sastry KJ (1996). Expression of human immunodeficiency virus type I tat results in down-regulation of bcl-2 and induction of apoptosis in hematopoietic cells. Oncogene.

[42] Macho A (1999). Susceptibility of HIV-1-TAT transfected cells to undergo apoptosis. Biochemical mechanisms. Oncogene.

[43] Westendorp MO (1995). Sensitization of T cells to CD95-mediated apoptosis by HIV-1 Tat and gp120. Nature.

[44] Castedo M (2004). Cell death by mitotic catastrophe: a molecular definition. Oncogene.

[45] Crozet, C. *et al*. Inhibition of PrPSc formation by lentiviral gene transfer of PrP containing dominant negative mutations. *J Cell Sci* **117**, 5591–5597, jcs.01484 (2004). 10.1242/jcs.01484 PMC2062426 15494372

[46] Ory DS, Neugeboren BA, Mulligan RC (1996). A stable human-derived packaging cell line for production of high titer retrovirus/vesicular stomatitis virus G pseudotypes. Proc Natl Acad Sci USA.

[47] Naldini L (1996). *In vivo* gene delivery and stable transduction of nondividing cells by a lentiviral vector. Science.

