# Supplementary material for: Effect of HIV-1 Tat on the formation of the mitotic spindle by interaction with ribosomal protein S3
Source: Sci Rep. 2018 Jun 6;8:8680. doi: 10.1038/s41598-018-27008-w (PMC5989196; doi:10.1038/s41598-018-27008-w)

**Supplementary Information**

Effect of HIV-1 Tat on the formation of mitotic spindle by interaction with ribosomal protein S3

# Jiyoung Kim and Yeon-Soo Kim

**Supplementary Figure S1. Association of RPS3 with Tat.** HEK 293FT cells were transfected with the Tat expression construct and incubated for 16 h. Cytosolic fractions were separated and subjected to immunoprecipitation with an anti-Tat antibody, an anti-RPS3 antibody, or normal serum. Immunoprecipitates obtained using normal serum or anti-Tat antibody were washed with buffer containing 300 mM NaCl, while those obtained using an anti-RPS3 antibody were washed with buffer containing 100 mM NaCl. Bound proteins were analyzed by immunoblotting with anti-Tat, anti-RPS3, anti-RPS6, and anti-RPL26 antibodies.

**Supplementary Figure S2. RPS3, but not RPL26 or RPS6, accumulates in the nucleus in the presence of Tat.** Fluorescence microscopy images of Tat-transfected 293FT cells immunostained with anti-Tat and anti-RPL26, anti-RPS3, or anti-RPS6 antibodies are shown. 293FT cells were transfected with the Tat expression construct, incubated for 24 h, and stained with anti-Tat and anti-RPL26, anti-RPS3, or anti-RPS6 antibodies.

**Supplementary Figure S3. shRPS3 efficiently knocks down RPS3 and inhibits cell growth.** (A) shRNA-mediated knockdown of RPS3. Four shRNA constructs targeting human RPS3 (arbitrarily named shRPS3-1, shRPS3-2, shRPS3-3, and shRPS3-4) and a control plasmid were purchased from SIGMA (SIGMA Mission shRNA, SHCLNG-NM_001005). shRPS3-4 was used in subsequent studies. 293FT cells were transduced with a lentivirus expressing the shRPS3 construct and incubated for 3 days. Whole cell lysates were prepared and analyzed by immunoblotting with antibodies against α-tubulin and RPS3. (B) Knockdown of RPS3 inhibits growth of 293FT cells. 293FT cells were transduced with a lentivirus expressing the shRPS3 construct and incubated for 6 days. (C) MTT assay using RPS3-knockdown cells. HEK 293FT cells (6 × 10^3^ cells/well in a 96-well plate) were transduced with a lentivirus expressing the shRPS3 construct and incubated for 3 days. Thereafter, 20 µl of MTT was added to each well and incubated for 4 h. The culture supernatant was carefully removed using a micropipette and 200 μl of DMSO was added to each well. The optical density at 560 nm was measured and the optical density at 670 nm was subtracted as background. Data represent the mean ± SEM. Statistical analysis was performed using a one-way ANOVA. P = 0.0098. (D) Nuclear localization of Tat in RPS3-knockdown cells. 293FT cells were transduced with shRPS3 and transfected with the Tat expression construct the following day. Cells were incubated for a further 2 days and immunostained with anti-Tat and anti-RPS3 antibodies. (E) Aberrant nuclei in RPS3-knockdown cells. 293FT cells were transduced with the control virus or shRPS3 and incubated for 6 days. Cells were then stained with DAPI.

**Supplementary Figure S4. RPS3, but not RPS6, localizes in the mitotic spindle.** (A) Staining of HeLa cells with an anti-RPS3 antibody only. HeLa cells were transduced with the control virus or shRPS3 and incubated for 2 days. Cells were then immunostained with an anti-RPS3 antibody. (B) mCherry-RPS3 localizes in the mitotic spindle. HeLa cells were transfected with pmCherry-rps3 and incubated for 20 h. Cells were immunostained with an anti-α-tubulin antibody. (C) RPS6 does not localize in the mitotic spindle. HeLa cells were immunostained with anti-RPS3 and anti-RPS6 antibodies.

**Supplementary Figure S5. Construction of RPS3mt**. (A) Nucleotide sequences of wild-type and mutant RPS3 proteins that was designed to specifically knockdown wild-type RPS3, but not the mutant proteins by the shRPS3-4. Red capital letters indicate nucleotide mutations. (B) Selective knockdown of wild-type RPS3 protein expression by shRPS3-4. pmCherry-rps3 and pmCherry-rps3mts were transfected into HeLa cells that were transduced with either the mock virus or shRPS3-4. Cells were further incubated for 2 days, and expression of wild-type or mutant mCherry-RPS3 proteins was examined under a fluorescence microscope.

**Supplementary Figure S6. Full-length blots of the data presented in Figure 1*A***. White dotted lines show where the blots were cropped.

**Supplementary Figure S7. Full-length blots of the data presented in Figure 1*B***. White dotted lines show where the blots were cropped.

**Supplementary Figure S8. Full-length blots of the data presented in Figure 1*C***. Black dotted lines show where the blots were cropped.

**Supplementary Figure S9. Full-length blots of the data presented in Figure 1*D***. White dotted lines show where the blots were cropped.

**Supplementary Figure S10. Interaction between RPS3 and α-tubulin.** (A) Full-length blot of the data presented in Figure 4*D*. Black dotted lines show where the blot was cropped. (B) Immunoprecipitation with an anti-RPS3 antibody. HeLa cells were treated with 100 ng/ml nocodazole, incubated for 16 h, resuspended in PBS containing 1% NP-40 and protease inhibitors, and incubated for 30 min at 4°C with rotation. Cell extracts were subjected to immunoprecipitation with an anti-RPS3 antibody. Bound proteins were analyzed by immunoblotting with anti-RPS3 and anti-α-tubulin antibodies.

Supplementary Figure S1


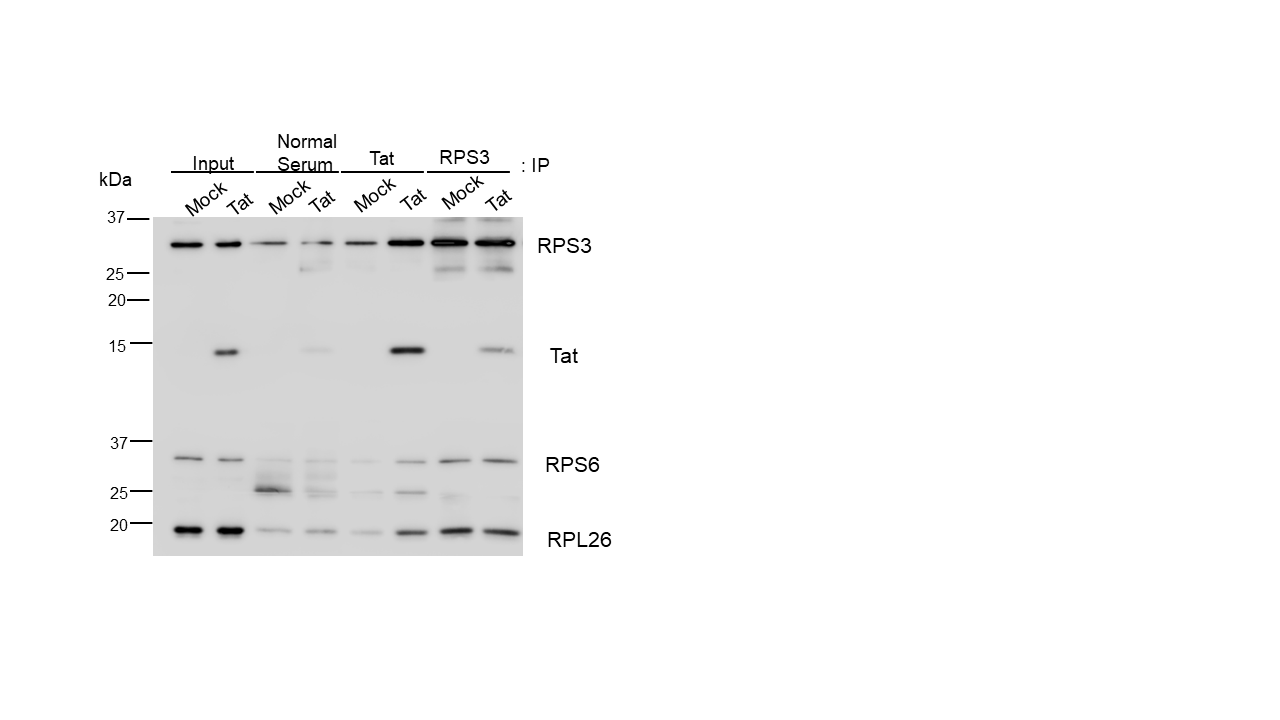


Supplementary Figure S2


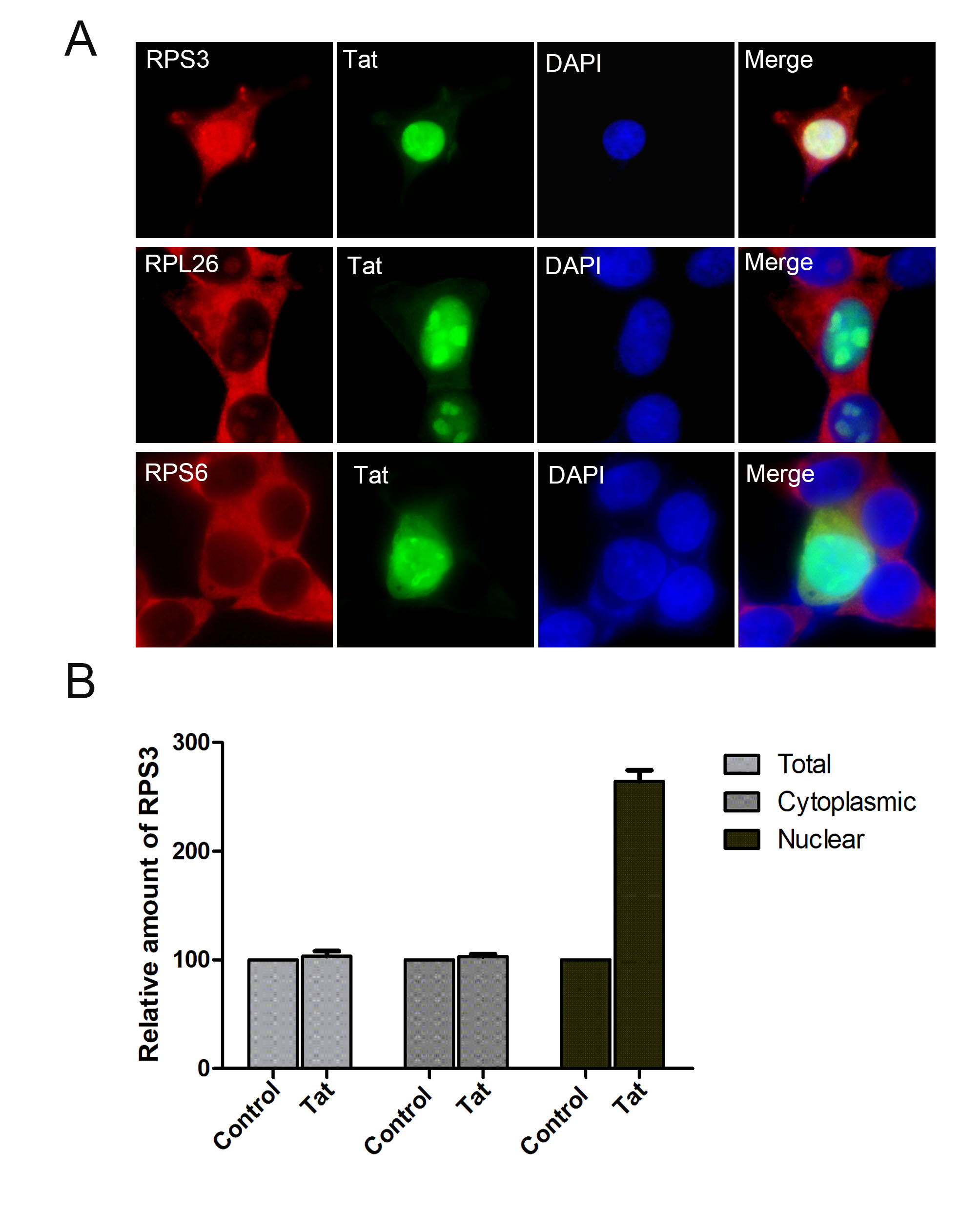


Supplementary Figure S3


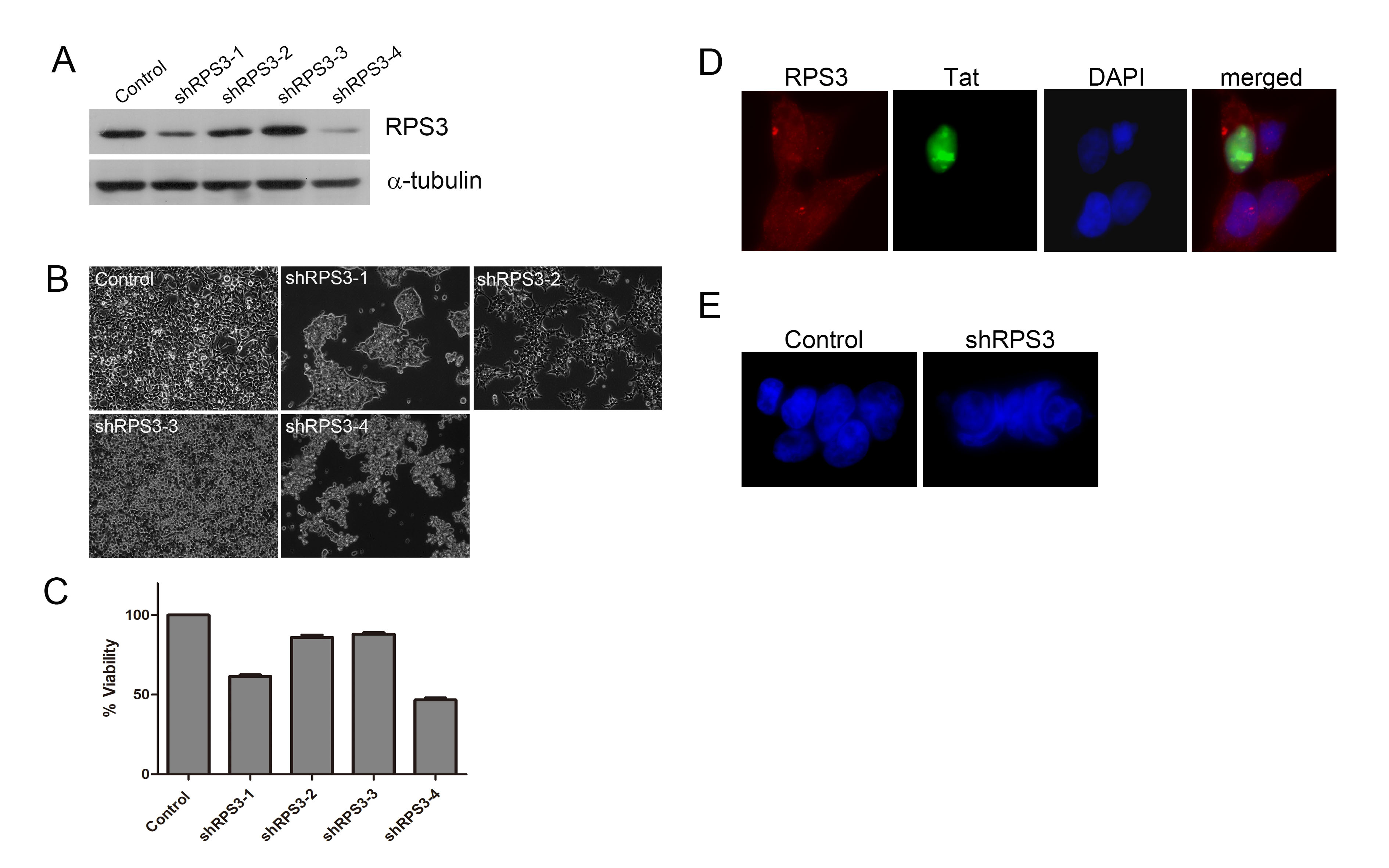


Supplementary Figure S4


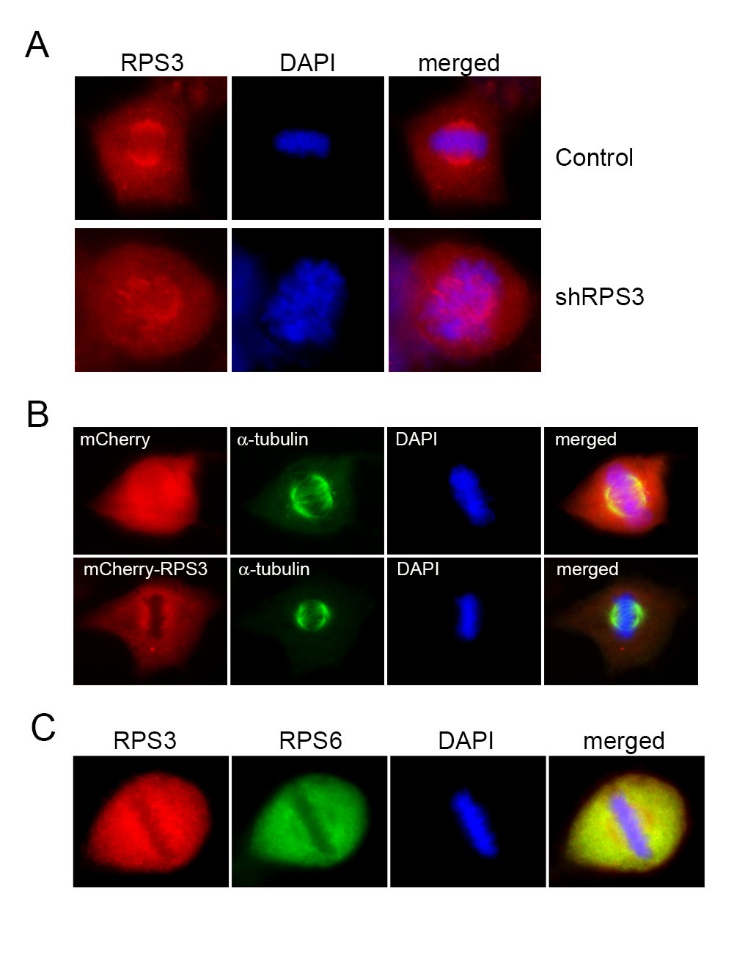


Supplementary Figure S5


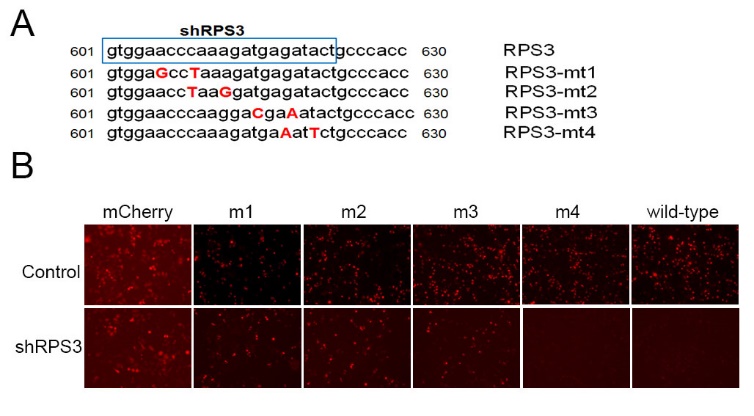


Supplementary Figure S6


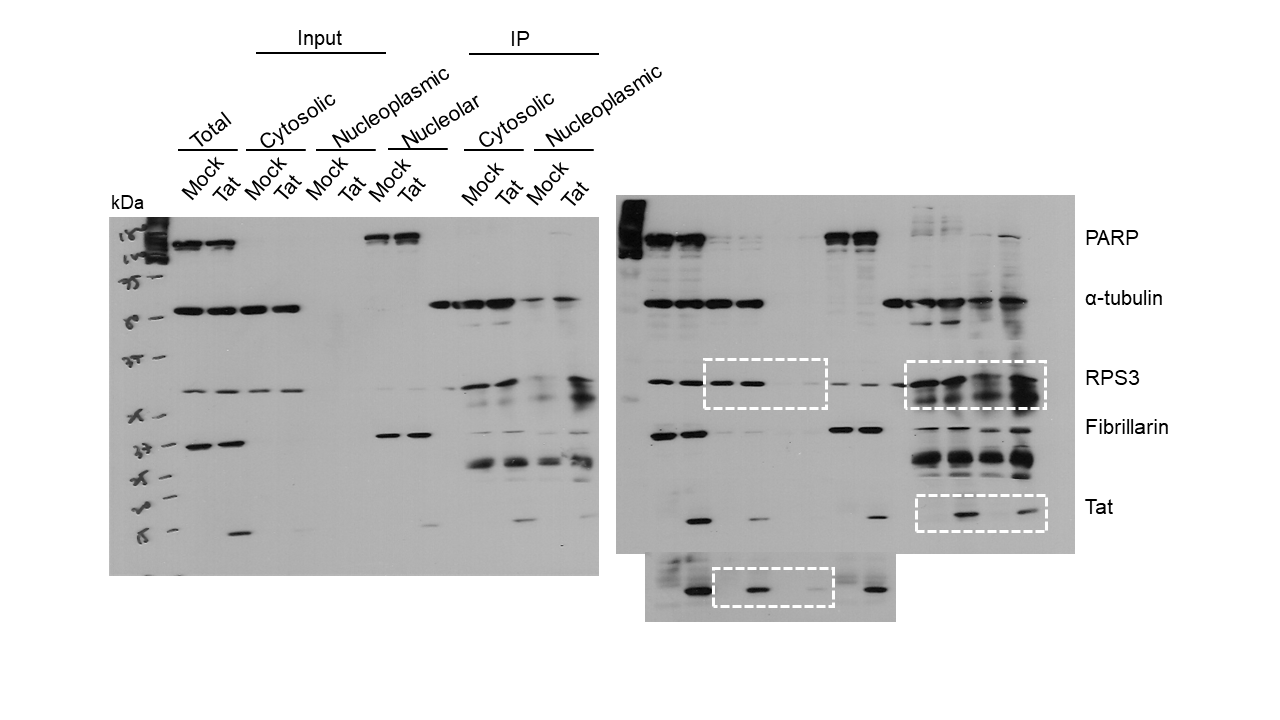


Supplementary Figure S7


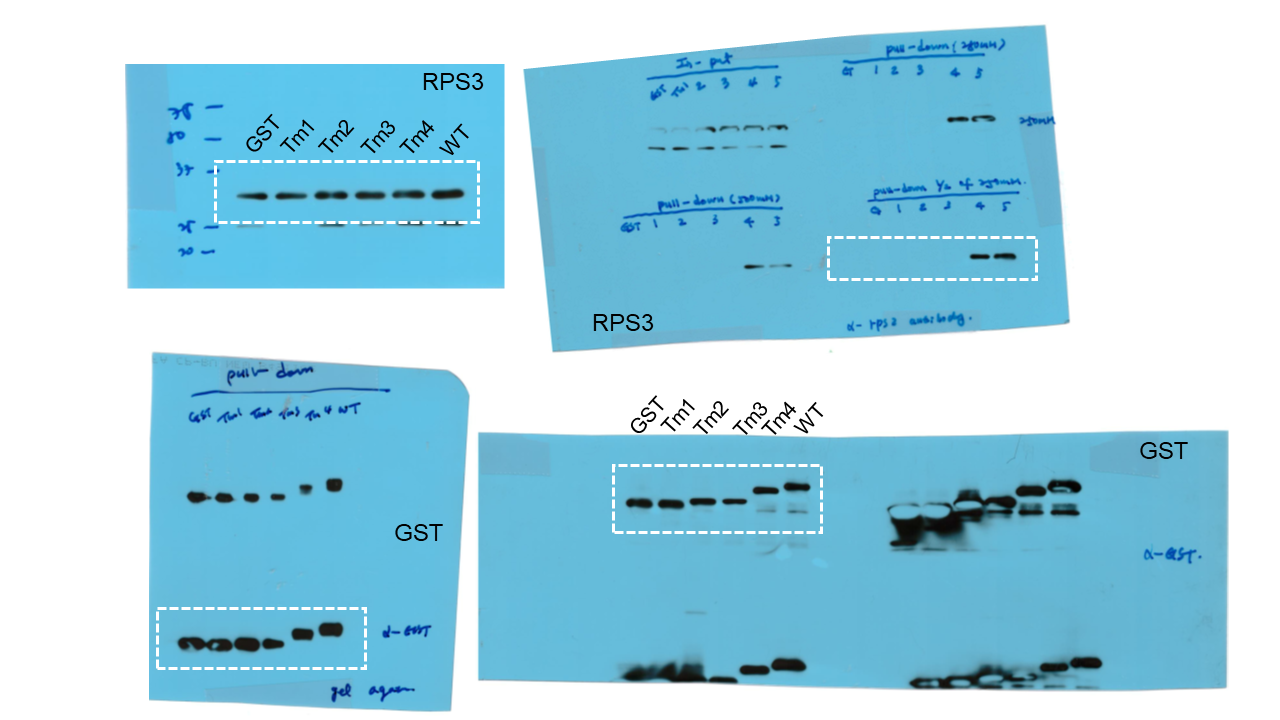


Supplementary Figure S8


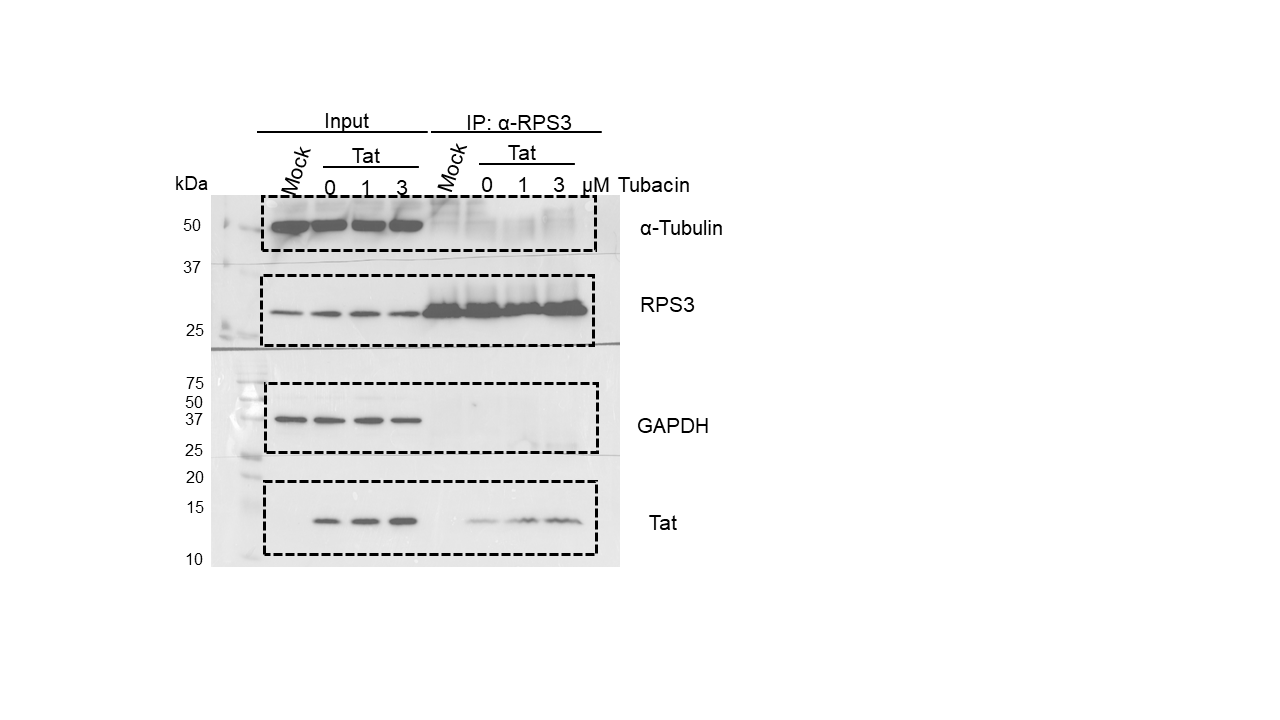


Supplementary Figure S9


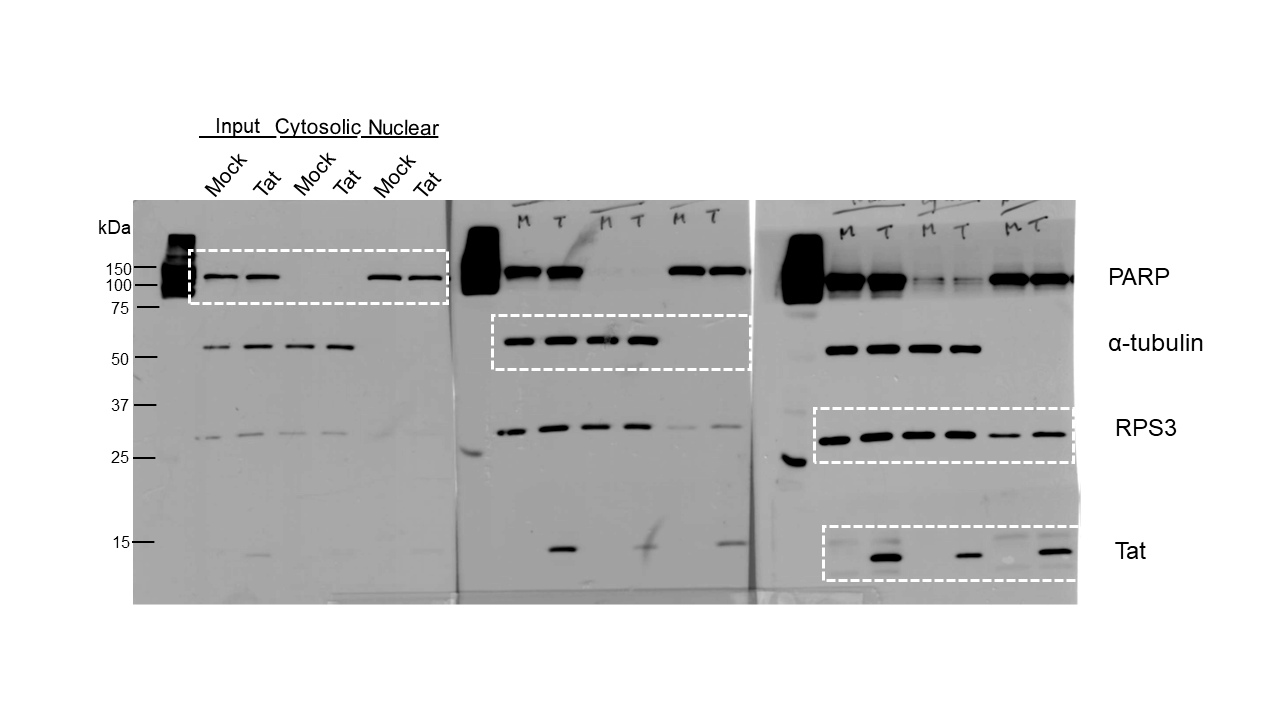


Supplementary Figure S10


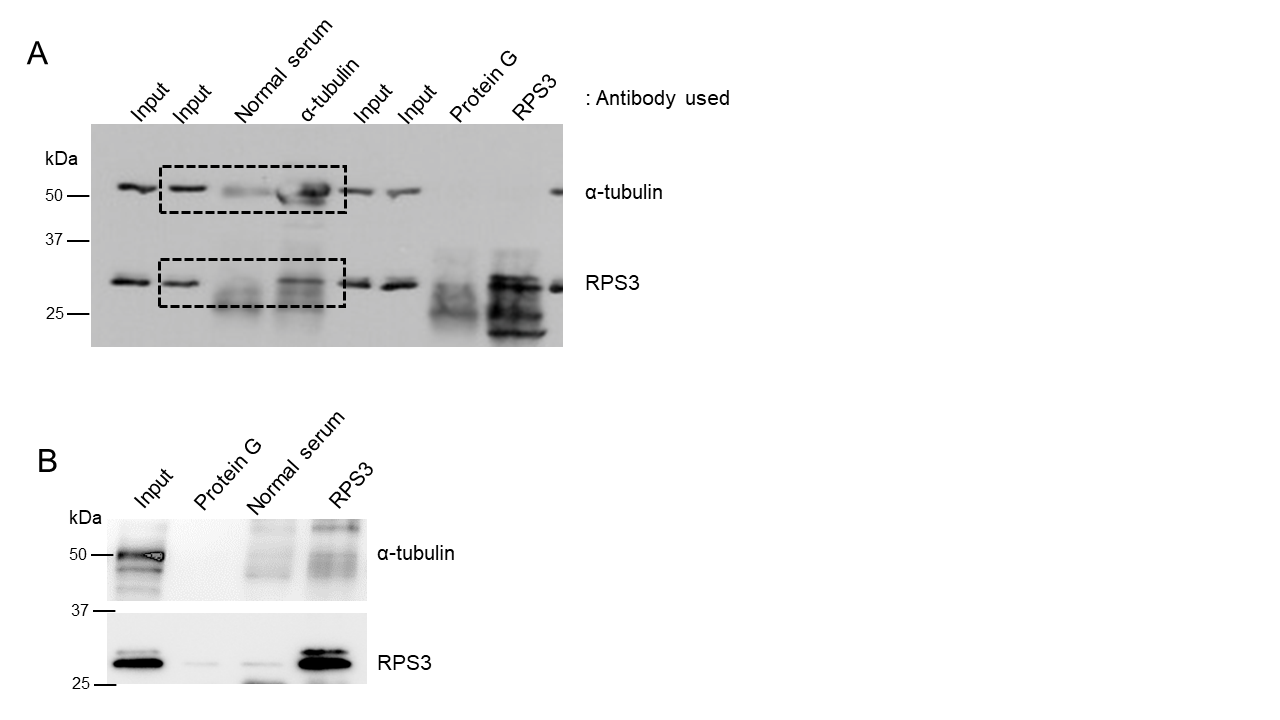

Supplement: Supplementary file 1 — Supplementary information [file 41598_2018_27008_MOESM1_ESM.doc]
